# Multi-Mode Nonlinear Ultrasonic Characterization of Fatigue Crack Evolution in GH4169 Nickel-Based Superalloy

**DOI:** 10.3390/ma19194151

**Published:** 2026-09-29

**Authors:** Jiajia Wang, Yaoyu He, Hangxing Shen, Jian Lu, Luran Li

**Affiliations:** 1 College of Science, Chang’an University, Xi’an 710072, China; 18655596104@163.com (Y.H.); 2023901071@chd.edu.cn (H.S.); 2024905158@chd.edu.cn (L.L.); 2 State Key Laboratory of Clean and Efficient Turbomachinery Power Equipment, Xi’an 710072, China; 3 AECC Aviation Power Co., Ltd., Xi’an 710021, China; nextlj@163.com

**Keywords:** nickel-based superalloys, fatigue, crack propagation, ultrasonic nonlinear parameter, nondestructive testing

## Abstract

**Highlights:**

**What are the main findings?**

- Nonlinear parameter changes before an optically detectable surface crack is observed.
- Non-monotonic three-stage evolution (increase–peak–decrease) of the nonlinear parameter is observed during fatigue.
- Different wave modes exhibit consistent non-monotonic trends despite different peak positions.

**What are the implications of the main findings?**

- Nonlinear ultrasonic measurements show sensitivity to early fatigue damage before the first optical detection of a surface crack.
- The nonlinear parameter reflects stage-dependent changes in the fatigue-damage and crack state rather than simply increasing with crack growth.
- Multi-mode nonlinear ultrasonic measurements provide complementary information on fatigue-damage evolution.

**Abstract:**

GH4169 nickel-based superalloy is widely used in critical aerospace components, where early fatigue damage detection is essential for structural safety. This study investigates the evolution of nonlinear ultrasonic responses during fatigue damage and crack propagation in GH4169 alloy through laser-fabricated controlled-crack specimens and interrupted fatigue tests on compact-tension specimens. Three wave modes—Rayleigh waves, normal-incidence longitudinal waves, and end-face-incidence longitudinal waves—were employed, and the results were compared with linear ultrasonic parameters. The controlled-crack experiments showed a crack-geometry dependence consistent with that predicted by the nonlinear spring model, with the normalized nonlinear parameter *β*′ exhibiting excellent linear correlations with *a*^2^ and 1/*b*^2^. The Rayleigh-wave nonlinear parameter exhibited a non-monotonic three-stage evolution: increasing during 6000–8000 cycles, peaking at 9000 cycles when an optically detectable surface crack was first observed, and decreasing thereafter. All three wave modes showed consistent overall non-monotonic trends despite different peak positions, while linear parameters remained essentially constant during the early fatigue stages. The observed three-stage evolution is interpreted as being associated with progressive changes in fatigue-induced microstructural damage, crack-related nonlinear interactions, and ultrasonic wave propagation as the crack grows. These findings demonstrate that multi-mode nonlinear ultrasonic techniques are sensitive to the evolution of fatigue damage and crack propagation and can provide complementary information for fatigue-damage characterization in aerospace components.

## 1. Introduction

Nickel-based superalloys are widely used in aero-engine hot-section components because of their excellent high-temperature strength, oxidation resistance, and microstructural stability [1,2,3]. Nevertheless, these components are subjected to complex cyclic thermomechanical loading during service, making fatigue damage and crack propagation critical factors affecting structural reliability and service life [4,5]. Recent studies on nickel-based superalloys have shown that fatigue damage is strongly associated with localized plastic deformation, crystallographic slip, microstructural heterogeneity, and the nucleation and early growth of small cracks [6,7]. In particular, short-crack behavior can differ substantially from that of long cracks described by conventional fracture-mechanics approaches, while crack initiation may consume a large fraction of the total fatigue life [8,9]. Therefore, reliable nondestructive evaluation methods capable of detecting fatigue-induced microstructural degradation and small cracks before they develop into readily detectable macroscopic defects are highly desirable.

Conventional linear ultrasonic testing has been extensively employed for structural inspection because of its robustness and practical applicability [10]. However, conventional linear parameters, including wave velocity, attenuation, reflection, and scattering, are generally more sensitive to relatively large geometric discontinuities than to distributed microstructural damage and partially closed microcracks [11]. In contrast, nonlinear ultrasonic techniques exploit the distortion of finite-amplitude acoustic waves caused by material or defect-induced nonlinearities, providing enhanced sensitivity to early-stage damage [12,13]. Among these approaches, second-harmonic generation is particularly attractive because the acoustic nonlinearity parameter can be derived from the amplitudes of the fundamental and second-harmonic components [14]. Previous studies have demonstrated that nonlinear ultrasonic parameters are sensitive to dislocation structures, plastic deformation, precipitate evolution, microvoids, and fatigue-induced microcracks [15,16]. For example, nonlinear ultrasonic measurements have successfully characterized fatigue damage in nickel-based superalloys, pearlitic steels, aluminum alloys, and other metallic materials [17,18,19]. More recent investigations have extended nonlinear ultrasonic characterization to nickel-based single crystal superalloys, in which the nonlinear parameter exhibited substantially higher sensitivity to early tensile plastic damage than conventional linear ultrasonic parameters [14]. Similarly, nonlinear ultrasonic measurements have been used to evaluate thermal degradation of nickel-based superalloys through their sensitivity to microstructural evolution and associated mechanical-property degradation [20]. These developments demonstrate the considerable potential of nonlinear ultrasonics for damage characterization in nickel-based superalloys.

The nonlinear ultrasonic response associated with fatigue-induced cracks may arise not only from intrinsic material nonlinearity but also from contact nonlinearity between opposing crack faces, with the latter representing an important source of crack-related acoustic nonlinearity. When a partially closed crack is subjected to finite-amplitude ultrasonic excitation, the two faces of the crack undergo periodic opening and closure—the so-called “breathing” behavior—giving rise to pronounced contact acoustic nonlinearity (CAN) and higher-order harmonic generation [21,22,23]. Richardson [21] first established a physical model that theoretically described the harmonic generation phenomenon arising from the periodic opening and closure of unbonded interfaces. Building upon this foundation, subsequent researchers developed nonlinear models incorporating the relationship between interface stiffness and contact pressure to describe the nonlinear response of rough contact interfaces [24]. Experimental studies have further demonstrated that the second-harmonic amplitude at contact interfaces is closely related to the contact state and surface roughness [25,26]. Aleshin and Van Den Abeele [27] further advanced this theoretical framework by incorporating the geometric characteristics and orientation distributions of rough microcracks and establishing quantitative relationships between crack morphology and acoustic nonlinearity. Ruiz and Kim [28] systematically investigated the combined effects of elastoplastic hysteresis, surface roughness, and adhesive forces on contact acoustic nonlinearity, revealing that clapping of lightly contacting cracks dominates the nonlinear response at low contact pressures, whereas elastoplastic nonlinear spring behavior prevails at high pressures. These theoretical and experimental advances provide an important physical basis for interpreting the nonlinear ultrasonic response of fatigue-damaged materials.

Based on this above-mentioned theoretical framework, the contact acoustic nonlinearity mechanism has been increasingly applied to the detection and characterization of closed and partially closed cracks, leading to the development of various inspection methods based on Rayleigh waves, Lamb waves, bulk waves, and nonlinear guided waves [29,30]. Numerous experimental studies have further demonstrated the applicability of nonlinear ultrasonic techniques for characterizing fatigue damage and crack propagation in metallic materials. Kim et al. [31] established a repeatable experimental procedure for monitoring fatigue damage in a nickel-base superalloy using the acoustic nonlinearity parameter *β*, demonstrating its sensitivity to fatigue-induced material degradation at an early stage. For GH4169 superalloy, Yan et al. [32] further reported that the second- and third-order nonlinear parameters exhibited a characteristic non-monotonic evolution with fatigue life, indicating that the nonlinear response was closely associated with the progressive development of fatigue damage. With increasing attention to crack-related nonlinearity, Wang et al. [33] applied nonlinear ultrasonic guided waves to characterize fatigue cracks in metallic plates and showed that the nonlinear response was sensitive to crack growth and depended on factors such as excitation frequency and the relative orientation between the wave propagation direction and the crack. More recently, Xu et al. [34] developed a three-dimensional analytical framework to describe second-harmonic generation induced by the breathing behavior of a non-penetrating fatigue crack and experimentally monitored the initiation and subsequent growth of a real fatigue crack. Lee and Lu [35] further investigated fatigue-crack detection under vibration using second-harmonic guided waves and demonstrated that variations in contact acoustic nonlinearity could be associated with crack growth under dynamic loading conditions. These studies have progressively extended nonlinear ultrasonic testing from the characterization of distributed fatigue damage to the monitoring of fatigue-crack evolution, while also highlighting the influence of crack geometry, wave mode, and loading conditions on the measured nonlinear response.

The objective of this study is to clarify the relationship between microcrack geometry and the nonlinear ultrasonic response and to characterize the evolution of fatigue damage and crack propagation in GH4169 nickel-based superalloy using multi-mode nonlinear ultrasonic measurements. A nonlinear contact-spring model is first employed to describe the interaction of rough microcracks under finite-amplitude ultrasonic excitation and to derive the dependence of the second-order nonlinear parameter on crack geometry. Laser-fabricated cracks with systematically varied lengths and widths are then introduced into GH4169 specimens by laser processing, and Rayleigh-wave second-harmonic measurements are performed to examine the consistency between the experimentally observed crack-geometry dependence and that predicted by the model. Subsequently, interrupted fatigue tests are conducted on CT specimens, and both Rayleigh-wave and longitudinal-wave measurements are employed to characterize the evolution of the nonlinear ultrasonic response during fatigue damage accumulation and crack propagation. Optical observations are used to identify the corresponding crack-growth stages, while longitudinal-wave velocity and attenuation are measured for comparison with the nonlinear ultrasonic response. The results provide insight into the relationship among crack geometry, nonlinear ultrasonic response, and fatigue crack evolution and offer a physically interpretable framework for the nonlinear ultrasonic evaluation of fatigue damage in nickel-based superalloys.

## 2. Theoretical Framework

### 2.1. Nonlinear Ultrasonic Parameter

When an ultrasonic wave with a finite amplitude propagates through a nonlinear medium, the waveform is distorted during propagation, resulting in the generation of higher-order harmonic components. For a sinusoidal excitation with a fundamental frequency *f*, the nonlinear response contains harmonic components at 2*f*, 3*f*, and higher frequencies. In metallic materials, these nonlinear effects may originate from intrinsic lattice nonlinearity, dislocation structures, microstructural defects, and, particularly for damaged materials, the nonlinear interaction between crack surfaces. The present study focuses on the second-harmonic component because it provides a convenient and sensitive indicator of acoustic nonlinearity.

For a monochromatic excitation, the fundamental displacement can be expressed as:

(1) $$u_{1}=A_{1}\sin\left(\omega t\right),$$

where $A_{1}$ is the amplitude of the fundamental component and ω=2πf is the angular frequency. Owing to the nonlinear wave propagation, a second-harmonic component is generated at 2ω, and the received displacement can be approximately expressed as:

(2) $$u=A_{1}\sin\left(\omega t\right)+A_{2}\sin\left(2\omega t+\varphi \right),$$

where $A_{2}$ is the amplitude of the second harmonic and φ is the corresponding phase difference.

Under the condition of weakly nonlinear wave propagation, the amplitude of the second harmonic is proportional to the square of the fundamental-wave amplitude when the material properties, wave number and propagation distance are fixed. Accordingly, the relative second-order acoustic nonlinearity parameter is defined as:

(3) $$\beta =\frac{A_{2}}{A_{1}^{2}},$$

where $A_{1}$ and $A_{2}$ are the amplitudes of the fundamental and second-harmonic components, respectively, extracted from the frequency-domain spectrum of the received ultrasonic signal.

It should be noted that an undamaged metallic material is not perfectly linear. Crystal defects, lattice imperfections, dislocations, and other microstructural features can contribute to the intrinsic acoustic nonlinearity. Consequently, the absolute value of *β* does not necessarily provide a direct measure of fatigue damage. For this reason, the evolution of the nonlinear parameter relative to the initial material condition is more meaningful for the present fatigue experiments.

Accordingly, the normalized nonlinear parameter is defined as:

(4) $$\beta^{\prime }=\frac{\beta \left(N\right)}{\beta \left(0\right)}$$

where *N* is the number of fatigue cycles, $\beta \left(N\right)$ is the relative nonlinear parameter measured after *N* fatigue cycles and $\beta \left(0\right)$ is the corresponding value before fatigue loading.

The normalized parameter is used throughout this study to describe the relative evolution of ultrasonic nonlinearity during fatigue damage accumulation.

### 2.2. Microcrack Nonlinear Spring Model

The nonlinear ultrasonic response associated with a microcrack can arise from the nonlinear interaction between opposing crack faces. When an ultrasonic wave propagates through a cracked material, the crack interface does not necessarily remain completely open or closed. Instead, the cyclic tensile and compressive stresses associated with the ultrasonic wave can induce repeated opening and closure of the crack. This dynamic contact behavior can produce a nonlinear mechanical response at the crack interface and consequently contribute to the generation of higher-order harmonic components in the transmitted ultrasonic wave.

For an idealized crack consisting of two initially contacting planar surfaces, the periodic tensile–compressive loading generated by an ultrasonic wave normal to the crack interface can induce repeated separation and contact of the two surfaces. However, actual fatigue cracks generally possess irregular and rough fracture surfaces rather than perfectly smooth planar interfaces.

Aeshin [27] approximated rough microcracks as two-dimensional planar cracks with an elliptical geometry, as schematically illustrated in Figure 1. The spatial orientation of a microcrack can be uniquely defined by two spherical angular parameters φ and θ, which describe the orientation of its normal vector. Here, θ denotes the angle between the microcrack normal and the *z*-axis (the direction of the applied external load), and φ represents the azimuth angle between the projection of the microcrack normal onto the x–y plane and the *x*-axis. Accordingly, an orientation distribution function Ψ(φ,θ) can be employed to characterize the spatial distribution of microcracks with diverse orientations.

Under cyclic external loading, cracks may undergo periodic opening and closure. The average crack width is denoted as $a_{\theta \varphi }$, and its value at zero applied stress is denoted by *A*, as shown in Figure 1b.

Neglecting the effect of shear stress on the microcracks, the three-dimensional normal strain can be expressed as:

(5) $$\begin{array}{l} \varepsilon_{xx}=\gamma_{xx}+S_{v}\int_{0}^{2/\pi }d\theta \int_{0}^{\pi }d\phi \left(a_{\theta \phi }-A\right)\psi \left(\theta ,\phi \right)\sin^{3}\theta \cos^{2}\phi \\ \varepsilon_{yy}=\gamma_{yy}+S_{v}\int_{0}^{2/\pi }d\theta \int_{0}^{\pi }d\phi \left(a_{\theta \phi }-A\right)\psi \left(\theta ,\phi \right)\sin^{3}\theta \sin^{2}\phi \\ \varepsilon_{zz}=\gamma_{zz}+S_{v}\int_{0}^{2/\pi }d\theta \int_{0}^{\pi }d\phi \left(a_{\theta \phi }-A\right)\psi \left(\theta ,\phi \right),\cos^{2}\theta \sin\theta \end{array}$$

where $\gamma_{ii}$ denotes the tensor of the undamaged material, and $S\left(\nu \right)$ represents the total area of microcracks per unit volume of the material. Since $\varepsilon \left(zz\right)$ is independent of φ, the expression can be simplified as:

(6) $$\varepsilon_{zz}=\gamma_{zz}+S_{v}\int_{0}^{2/\pi }\left(a_{\theta }-A\right)\cos^{2}\theta \sin\theta d\theta ,$$

Substituting Equation (6) into the ultrasonic wave equation yields the nonlinear wave equation for a medium containing microcracks under one-dimensional conditions. However, a direct analytical solution of this equation is difficult to obtain. Therefore, a nonlinear spring model is employed to describe the mechanical response of the crack interface under external loading, as shown in Figure 1c.

Consider a metallic plate of length *L* and width *H*, containing an elliptical microcrack located at $x_{0}$ with major and minor axes *a* and *b*, respectively. A longitudinal wave with angular frequency ω is incident along the length direction of the plate (the x-direction), and the acoustic field excited in the metallic plate is expressed as:

(7) $$u\left(x\right)=U\left(x\right)\cos\omega t,$$

Under cyclic loading, the opposing surfaces of a microcrack may undergo repeated opening and closure, resulting in a stress-dependent variation in the crack opening. If the mechanical response of the crack interface is represented by a nonlinear spring [36], the internal force acting on the microcrack can be expressed a:

(8) $$\Delta \sigma =K\left(\xi \right)\xi ,$$

where $\xi =U_{+}-U_{-}$ represents the variation in crack width, $K_{\xi }$ stands for the stiffness coefficient, and its first-order Taylor series expansion reads:

(9) $$K\left(\xi \right)=K_{0}+K_{1}\xi ,$$

Assuming infinitesimal deformation on both sides of the microcrack, $K_{1}\ll K_{0}$ holds. Substituting Equation (9) into Equation (8), we obtain:

(10) $$\Delta \sigma =K_{0}\xi +K_{1}\xi^{2}+O\left(\xi^{3}\right),$$

where $K_{1}$ represents the nonlinear property arising from microcracks and acts as a quantitative indicator of material damage severity.

Subject to excitation by the ultrasonic signal *u*(*x*), the change in microcrack size takes the form:

(11) $$\xi =\frac{a}{b}\frac{\partial U}{\partial x}{|}_{x=x_{0}}\cos\omega t,$$

Substituting Equation (11) into Equation (10), the internal stress generated by the incident ultrasonic wave at the microcrack is expressed as:

(12) $$\Delta \sigma =K_{0}\frac{a}{b}{\left.\frac{\partial U}{\partial x}\right|}_{x=x_{0}}\cos\omega t+{\left.\frac{K_{1}a^{2}}{2b^{2}}{\left(\frac{\partial U}{\partial x}\right)}^{2}\right|}_{x=x_{0}}\cos2\omega t-{\left.\frac{K_{1}a^{2}}{2b^{2}}{\left(\frac{\partial U}{\partial x}\right)}^{2}\right|}_{x=x_{0}},$$

where the two terms on the right-hand side correspond to the nonlinear contributions arising from the nonlinear parameter $K_{1}$ and are defined respectively as:

(13) $$\Delta \sigma_{1}=\frac{K_{1}a^{2}}{2b^{2}}{\left(\frac{\partial U}{\partial x}\right)}^{2}\cos2\omega t,$$

(14) $$\Delta \sigma_{2}=\frac{K_{1}a^{2}}{2b^{2}}{\left(\frac{\partial U}{\partial x}\right)}^{2},$$

As $∆\sigma_{2}$ constitutes a constant term, only $∆\sigma_{1}$ is taken into account. Let *S* be the microcrack area; then the internal force produced by the internal stress on the microcrack reads:

(15) $$F_{1}=S\Delta \sigma_{1},$$

Thus, $F_{1}$ takes the form:

(16) $$F_{1}=S\cdot \frac{K_{1}a^{2}}{2b^{2}}{\left(\frac{\partial U}{\partial x}\right)}^{2}\cos2\omega t,$$

Let $U\left(x\right)=A_{1}coskx$, in which *k* is the wave number, and consider the boundary conditions:

(17) $$U\left(x=x_{0}^{-}\right)=U\left(x=x_{0}^{+}\right),$$

(18) $${\left.\sigma \right|}_{x=x_{0}^{+}}-{\left.\sigma \right|}_{x=x_{0}^{-}}=\frac{2F}{S},$$

Accordingly, the second harmonic of the received ultrasonic signal $U_{0}\left(x\right)$ reads:

(19) $$U_{0}(x)=\frac{1-v^{2}}{E}\cdot \frac{K_{1}a^{2}A_{1}^{2}k}{b^{2}}\left(\frac{\cos kx_{0}}{\sin kL}\sin kx_{0}\right)\cos k\left(L-x\right),$$

Therefore, the amplitude of the second harmonic of the received signal reads:

(20) $$A_{2}=\frac{1-v^{2}}{E}\cdot \frac{K_{1}a^{2}A_{1}^{2}k}{b^{2}},$$

Accordingly, the definition of the second-order ultrasonic nonlinearity parameter is given by:

(21) $$\beta =\frac{A_{2}}{A_{1}^{2}}=\frac{1-v^{2}}{E}\cdot \frac{K_{1}a^{2}A_{1}^{2}k}{b^{2}},$$

The nonlinear spring model therefore establishes a theoretical relationship between microcrack geometry and the second-harmonic ultrasonic response. Specifically, the model predicts that the theoretical nonlinear parameter is proportional to the square of the crack length and inversely proportional to the square of the crack width, i.e., $\beta \propto a^{2}/b^{2}$. This relationship provides the theoretical basis for examining the experimentally observed dependence of the relative nonlinear parameter $\beta^{\prime }$ on crack length and width.

## 3. Materials and Methods

### 3.1. Material and Specimen Preparation

The material investigated in this study was GH4169 nickel-based superalloy, a precipitation-strengthened Ni–Cr–Fe-based superalloy widely used in critical components of aerospace propulsion systems because of its high strength, oxidation and corrosion resistance, and good long-term microstructural stability [37]. The material was obtained from a turbine-disk component and was subjected to a standard heat-treatment procedure prior to specimen preparation: (950–980) °C × 1 h/AC + 720 °C × 8 h/FC → 620 °C × 8 h/AC, where AC and FC denote air cooling and furnace cooling, respectively. The nominal chemical composition of the GH4169 alloy is listed in Table 1. The main mechanical-property parameters of the material are listed in Table 2 [38].

Two types of specimens were prepared for different experimental purposes, as schematically illustrated in Figure 2. Flat-plate specimens containing laser-fabricated cracks were used to experimentally examine the dependence of the nonlinear ultrasonic response on crack geometry and its consistency with the crack-geometry dependence predicted by the nonlinear spring model developed in Section 2.2. Compact-tension (CT) specimens were employed for interrupted fatigue crack growth experiments to investigate the response of nonlinear and linear ultrasonic parameters during fatigue damage accumulation and crack propagation.

For the controlled-crack experiments, GH4169 plates were machined into rectangular specimens with dimensions of 80 mm × 14 mm × 1.5 mm, as shown in Figure 2a. Artificial cracks with controllable geometric dimensions were fabricated at the center of the specimens using the Micro-Drill100 femtosecond laser processing system (Xi’an CAS Microstar Photonics Co., Ltd., Xi’an, China). The laser processing parameters were adjusted to ensure that all artificial cracks penetrated through the specimen thickness. The laser-fabricated crack surfaces exhibited a surface roughness of less than 0.2 μm. By independently varying the crack length and width, the influence of crack geometry on the nonlinear ultrasonic response could be experimentally isolated. The geometrical parameters of the laser-fabricated cracks used in the two experimental series are summarized in Table 3. In the first series, the crack width was fixed at 100 μm while the crack length was varied from 300 to 500 μm in 50 μm increments; in the second series, the crack length was fixed at 320 μm while the crack width was varied from 100 to 180 μm in 20 μm increments.

For the fatigue experiments, compact-tension specimens were machined from the same heat-treated GH4169 material, with detailed geometric dimensions shown in Figure 2b. The CT specimens had a width of W = 40 mm and a thickness of B = 8 mm. The specimen geometry was designed with reference to HB 7680-2000 [39]. A machined V-notch with an opening angle of 60° was introduced into each specimen. The initial notch length, $a_{0}$, measured from the load line to the notch tip, was 8 mm, corresponding to $a_{0}/W=0.20$. No fatigue pre-cracking was performed before the fatigue tests. All fatigue specimens were prepared from the same material batch and subjected to the same heat-treatment condition, thereby reducing specimen-to-specimen variations in microstructure and mechanical properties.

### 3.2. Interrupted Fatigue Testing

A total of nine CT specimens were prepared for the fatigue experiments. All CT specimens were machined from the same GH4169 parent material using identical specimen geometry and preparation procedures and were subjected to the same fatigue loading conditions to minimize potential scatter in the fatigue tests. One CT specimen was first subjected to a complete fatigue test to determine the overall fatigue-life range and crack-growth behavior, which provided the basis for selecting the subsequent interruption points. Of the remaining eight CT specimens, one was retained without fatigue loading as the 0-cycle reference specimen, while the other seven specimens were independently subjected to 6000, 7000, 8000, 9000, 10,000, 11,000, and 12,000 fatigue cycles, respectively.

Fatigue tests were conducted at room temperature using an Instron 8802 servo-hydraulic fatigue testing machine (Instron Corporation, Norwood, MA, USA), as shown in Figure 3. The tests were performed under load control using a sinusoidal waveform at a frequency of 3 Hz. The maximum applied load was $P_{max}=14.4\;k$N, with a load ratio of $R=P_{min}/P_{max}=0.1$, corresponding to a minimum load of $P_{min}=1.44\;k$N and a load range of ΔP=12.96 kN. During fatigue loading, time, applied load, and displacement were continuously recorded at a sampling frequency of 200 Hz. Based on the applied load range and the optically measured surface crack length, the stress-intensity-factor range, ΔK, during the optically detectable crack-growth stage was estimated according to Refs. [39,40].

After each fatigue test, the specimen was loaded to the maximum fatigue load and maintained at this load during image acquisition. The surface crack morphology was observed using the optical imaging system of a VIC-3D digital image correlation (DIC) system (Correlated Solutions, Inc., Irmo, SC, USA). The acquired images were used to characterize the evolution of surface crack morphology and to measure the crack extension at different fatigue-cycle conditions.

### 3.3. Nonlinear Ultrasonic Characterization

The finite-amplitude ultrasonic method was employed to characterize the nonlinear ultrasonic response of the GH4169 specimens. The amplitudes of the fundamental and second-harmonic components of the received signals were extracted and used to calculate the nonlinear parameter *β* according to Equation (3).

Rayleigh waves, longitudinal waves, and Lamb waves are commonly employed for nonlinear ultrasonic characterization, with different wave modes requiring different transducer configurations and propagation paths. In the same-side configuration, the transmitting and receiving transducers are positioned on the same surface of the specimen, allowing Rayleigh or Lamb waves to propagate along the specimen surface. In contrast, the opposite-side configuration places the transmitting and receiving transducers coaxially on opposite sides of the specimen, enabling longitudinal waves to propagate through the specimen thickness.

In the present study, Rayleigh waves were employed for nonlinear ultrasonic characterization of both the laser-fabricated crack specimens and the CT specimens subjected to interrupted fatigue loading. In addition, longitudinal-wave measurements were performed on the CT specimens to provide complementary characterization of the evolution of the nonlinear ultrasonic response during fatigue damage and crack propagation. For each specimen and each ultrasonic measurement configuration, five repeated measurements were performed, with the transducers removed and re-coupled before each measurement. Unless otherwise specified, the reported data points represent the mean values of the five repeated measurements, and the error bars indicate ±1 standard deviation (SD).

#### 3.3.1. Nonlinear Ultrasonic Measurement Using Rayleigh Waves

The Rayleigh-wave ultrasonic measurement system, as schematically illustrated in Figure 4a, was employed to characterize the laser-fabricated crack specimens and examine the dependence of the nonlinear ultrasonic response on the controlled crack geometry.

High-amplitude ultrasonic signals were generated using a RIETC high-energy nonlinear ultrasonic system (RITEC Inc., Warwick, RI, USA). The excitation signal was transmitted through a 50 Ω impedance, an adjustable attenuator, and a low-pass filter before being applied to the transmitting transducer. After propagating through the specimen, the ultrasonic signal was received by the second-harmonic receiving transducer. The generated higher-harmonic components were subsequently processed using a high-pass filter and a preamplifier before being acquired by the measurement system. The received signals were finally transformed into the frequency domain using fast Fourier transformation (FFT), from which the amplitudes of the fundamental and second-harmonic components, $A_{1}$ and $A_{2}$, respectively, were extracted for the calculation of *β*.

Rayleigh-wave ultrasonic transducers with an incidence angle of 72° were employed in the experiments. The excitation signal was a 25-cycle Hanning-windowed sinusoidal burst with a centre frequency of 2.5 MHz. An ultrasonic transducer with a centre frequency of 5 MHz was adopted to receive the fundamental and second-harmonic signals. The transducers were placed on the centreline of the specimen, with the transmitting and receiving transducers located on opposite sides of the laser-fabricated crack, and the distance between them was 30 mm, as shown in Figure 4a. A custom fixture was designed to guarantee precise alignment of the transmitting and receiving transducers and maintain stable coupling between the transducers and the specimen. Glycerol was selected as the coupling agent.

Subsequently, the same Rayleigh-wave measurement configuration was employed for the interrupted fatigue specimens to monitor the evolution of the nonlinear ultrasonic response during fatigue crack propagation. The ultrasonic transducers were positioned at the notch root of the CT specimen during measurement, as shown in Figure 4c.

#### 3.3.2. Nonlinear Ultrasonic Measurement Using Longitudinal Waves

The longitudinal-wave nonlinear ultrasonic measurement system illustrated in Figure 4b was adopted to measure the longitudinal-wave nonlinear ultrasonic coefficients of interrupted-fatigue C(T) specimens at different fatigue cycles, so as to provide complementary characterization of the nonlinear ultrasonic response associated with fatigue damage and crack propagation. A longitudinal-wave transducer with a chip diameter of 8 mm and a centre frequency of 5 MHz was used for excitation. The transmitted signal was an eight-cycle Hanning-windowed sinusoidal burst, and a longitudinal-wave transducer with a centre frequency of 10 MHz was employed for signal reception. As shown in Figure 4c, the transducer was positioned at the tip of the V-shaped notch. Ultrasonic waves were normally incident from the top surface of the specimen, propagating through the specimen thickness and interacting with the fatigue crack plane.

Since the ultrasonic wave is approximately parallel to the crack plane under normal-incidence of longitudinal waves, as shown in Figure 5a, the wave modulation effect is relatively weak, which may result in an insignificant nonlinear effect. Therefore, supplementary longitudinal-wave nonlinear ultrasonic measurements were performed using the end-face-incidence configuration. During measurement, ultrasonic waves were introduced from the specimen end face, and the wave propagation direction was approximately perpendicular to the crack plane, as shown in Figure 5b. For each interrupted specimen, the Rayleigh-wave and normal-incidence longitudinal-wave measurements were first performed on the intact CT specimen. Subsequently, a small block containing the crack region was sectioned from the CT specimen and labeled according to its corresponding fatigue-cycle condition and ultrasonic incidence direction. Specimens with dimensions of 8 mm × 8 mm × 8 mm were prepared for each target fatigue state, as shown in Figure 5c. The end-face-incidence longitudinal-wave measurement was then performed on the sectioned block, with the transmitting and receiving transducers coaxially positioned on the two opposite end faces. This measurement sequence was consistently applied to all fatigue-cycle conditions.

### 3.4. Linear Ultrasonic Characterization

Conventional linear ultrasonic testing methods generally detect defects through changes in ultrasonic reflection, scattering, amplitude, phase, or energy resulting from wave–defect interactions. For conventional defect detection based on reflected or scattered signals, relatively large discontinuities, such as open cracks and inclusions, can produce readily detectable ultrasonic responses because of differences in acoustic properties between the defect and the surrounding material. However, such conventional defect-detection approaches generally exhibit lower sensitivity to early-stage microstructural degradation and partially closed fatigue cracks.

In addition to conventional defect detection, linear ultrasonic parameters, including longitudinal-wave velocity and ultrasonic attenuation coefficient, have been widely used to characterize changes in material microstructure and mechanical properties. The propagation velocity of longitudinal waves is closely related to the elastic properties and density of the material and can be expressed as follows [41]:

(22) $$c_{l}=\sqrt{\frac{E\left(1-\nu \right)}{\rho \left(1+\nu \right)\left(1-2\nu \right)}},$$

where *E*, ρ and ν represent the elastic modulus, Poisson’s ratio, and density of the material, respectively. Therefore, variations in longitudinal-wave velocity may reflect changes in the effective elastic properties associated with fatigue damage, residual stress, and microstructural evolution. In addition, the ultrasonic attenuation coefficient is sensitive to wave scattering and energy dissipation associated with microstructural features and defects, such as cracks and dislocations, and can therefore provide complementary information regarding material degradation [42].

In this study, longitudinal-wave velocity and attenuation coefficient were measured on the interrupted CT specimens to provide linear ultrasonic references for comparison with the nonlinear ultrasonic response. The measurements were performed using the ultrasonic pulse-echo method. A longitudinal-wave ultrasonic transducer with a diameter of 8 mm and a center frequency of 5 MHz was employed. During the measurement, the transducer was coupled to the specimen surface using glycerol, and the ultrasonic signals were acquired under identical experimental conditions for all fatigue states.

The longitudinal-wave velocity was determined from the specimen thickness and the time interval between successive back-wall echoes according to:

(23) $$c_{l}=\frac{2B}{T},$$

where *B* is the specimen thickness and *T* represents the time interval between successive ultrasonic echoes.

The ultrasonic attenuation coefficient was calculated from the amplitudes of multiple back-wall echoes according to [43]:

(24) $$\alpha =\frac{20}{\left(x_{n}-x_{m}\right)}\mathrm{lg}\left(\frac{A_{m}}{A_{n}}\right),$$

where $A_{m}$ and $A_{n}$ are the amplitudes of the *m*-th and *n*-th back-wall echoes, respectively, and $x_{n}-x_{m}$ represents the corresponding propagation distance difference.

To minimize experimental uncertainty, the ultrasonic measurement system, transducer configuration, coupling procedure, and signal-processing parameters were kept unchanged for all specimens. Each fatigue state was measured repeatedly, and the average value was used as the representative longitudinal-wave velocity and attenuation coefficient for subsequent analysis. The obtained linear ultrasonic parameters were compared with the nonlinear ultrasonic parameter *β* to evaluate their respective sensitivities to fatigue-induced damage and crack propagation.

## 4. Results and Discussion

### 4.1. Crack-Geometry-Dependent Nonlinear Ultrasonic Response of Laser-Fabricated Cracks

#### 4.1.1. Reliability Verification of the Nonlinear Ultrasonic Measurement System

The nonlinear ultrasonic response of metallic materials is influenced not only by microstructural damage but also by the stability and sensitivity of the measurement system. Therefore, before investigating the relationship between crack geometry and nonlinear ultrasonic response, the reliability of the nonlinear ultrasonic measurement system was first evaluated using intact GH4169 specimens without artificial cracks.

The Rayleigh-wave nonlinear ultrasonic measurement system was evaluated through voltage and propagation-distance calibrations. For voltage calibration, the measurement distance was fixed at 30 mm, and the excitation voltage was increased from 50 V to 65 V in increments of 5 V increments. The square of the fundamental-wave amplitude was plotted against the second-harmonic amplitude on the vertical axis, as shown in Figure 6a.

For propagation-distance calibration, the initial transducer separation was set to 30 mm to minimize the influence of the transducer near-field region, and the distance between the transmitting and receiving transducers was subsequently increased in increments of 4 mm. A typical received time-domain signal is shown in Figure 6b. The signal amplitude decreases with increasing ultrasonic propagation distance. A fast Fourier transform (FFT) was then applied to the received signals to extract the amplitudes of the fundamental and second-harmonic components. The variation in the nonlinear parameter with propagation distance is shown in Figure 6c and exhibits an approximately linear trend, consistent with the cumulative nature of second-harmonic generation under the present measurement conditions. The favorable linear relationships obtained from the voltage and propagation-distance calibrations confirm the expected behavior of the nonlinear ultrasonic measurement system and support the reliability of the subsequent nonlinear ultrasonic measurements.

For the longitudinal-wave nonlinear ultrasonic measurement system, only voltage calibration was performed because the propagation distance was fixed by the specimen thickness. The same undamaged specimen was used, and the excitation voltage was increased from 40 V to 60 V in increments of 5 V. The relationship between the second-harmonic amplitude and the square of the fundamental-wave amplitude is shown in Figure 6d and exhibits a clear linear trend. This result confirms the expected relationship between the fundamental and second-harmonic amplitudes and supports the reliability of the longitudinal-wave nonlinear ultrasonic measurements.

#### 4.1.2. Effects of Crack Length and Width on the Nonlinear Ultrasonic Response

After confirming the reliability of the second-harmonic measurement system, laser-fabricated crack specimens with controlled geometrical parameters were investigated to examine the dependence of the nonlinear ultrasonic response on crack geometry. As described in Section 2.2, the nonlinear spring model establishes a theoretical relationship between crack geometry and the second-order nonlinear parameter, predicting that the nonlinear response increases with crack length and decreases with crack opening width. The laser-fabricated specimens therefore provide controlled crack geometries for experimentally examining whether the measured nonlinear response exhibits the crack-size dependence predicted by the theoretical model.

Figure 7a presents the variation in the normalized nonlinear parameter *β* with crack length at a fixed crack width of 100 μm. As the crack length increases from 300 to 500 μm, *β* exhibits a continuous increasing trend. This result indicates that the measured nonlinear ultrasonic response is sensitive to crack length under otherwise identical experimental conditions. In contrast, when the crack length is fixed at 320 μm, *β* decreases systematically as the crack width increases from 100 to 180 μm, as shown in Figure 7b. Thus, the experimental results reveal opposite dependencies of the nonlinear response on crack length and crack opening width: an increase in crack length enhances the measured nonlinear response, whereas an increase in crack opening width reduces it.

To further compare the experimentally observed crack-geometry dependence with the theoretical relationship derived in Section 2.2, the experimental data were replotted as *β* versus $a^{2}$ and *β* versus $b^{2}$, respectively, according to Equation (21). As shown in Figure 7c, *β* exhibits a strong linear correlation with $a^{2}$, with a coefficient of determination of R^2^ = 0.99. Similarly, Figure 7d shows an approximately linear relationship between *β* and 1/*b*^2^, with R^2^ = 0.97. These results demonstrate that, within the investigated range of crack geometries, the experimentally observed dependence of the nonlinear parameter on crack length and width is consistent with the trend predicted by Equation (21).

It should be noted that the laser-fabricated cracks provide artificial discontinuities with well-controlled geometrical parameters, while their interfacial conditions may differ from those of naturally developed fatigue cracks. Therefore, the present analysis primarily focuses on the experimentally observed dependence of the nonlinear response on crack geometry and its correspondence with the theoretical prediction. The results show that *β* increases with crack length and decreases with crack opening width, in agreement with the trend predicted by the nonlinear spring model. Meanwhile, the measured nonlinear response represents the overall interaction between the ultrasonic wave and the artificial crack; in addition to crack-interface effects, wave-propagation and scattering effects associated with the crack geometry may also contribute to the measured response.

Overall, the controlled-crack experiments reveal a clear dependence of the nonlinear ultrasonic response on crack geometry. In particular, the good correlations of *β* with $a^{2}$ and 1/b^2^ demonstrate that the experimental results are consistent with the crack-geometry dependence described by the theoretical model. These observations provide experimental support for the important roles of crack size and opening condition in governing the nonlinear ultrasonic response and establish a theoretical and experimental basis for interpreting the subsequent evolution of nonlinear ultrasonic signals during fatigue crack development.

### 4.2. Evolution of Nonlinear Ultrasonic Response During Fatigue Crack Propagation

#### 4.2.1. Optical Observation of Fatigue Crack Morphology

To systematically investigate the evolution of nonlinear ultrasonic responses throughout the entire process of fatigue damage accumulation and subsequent macroscopic crack propagation, a series of interrupted fatigue tests were conducted on compact tension (CT) specimens. These specimens were subjected to varying numbers of fatigue cycles, after which they were examined using the optical imaging system of a VIC-3D digital image correlation (DIC) system (Correlated Solutions, Inc., USA) and nonlinear ultrasonic measurement techniques. For optical observation, each specimen was loaded to the maximum load used in the fatigue test and maintained at this load during image acquisition. This loading condition kept the fatigue crack open and facilitated observation of the surface crack morphology, as the crack faces tended to close after unloading. The surface images acquired using the VIC-3D system were used to monitor the evolution of fatigue crack morphology at different fatigue stages.

Figure 8 displays representative optical micrographs obtained at four selected fatigue cycle stages, namely 6000, 8000, 9000, and 12,000 cycles. At the early stages of 6000 and 8000 cycles, no optically detectable surface crack was observed in the vicinity of the notch root, suggesting that the material remained in a pre-critical damage regime where microstructural alterations had not yet coalesced into a detectable geometric discontinuity. In contrast, at 9000 cycles, a surface crack with a length of approximately 0.248 mm became clearly visible near the notch root, indicating the transition to an optically detectable crack-growth stage. As cyclic loading proceeded further, the crack propagated progressively away from the notch region, and by 12,000 cycles. The quantitatively measured surface crack extensions during this optically detectable crack-growth stage are summarized in Table 4. The crack extension increased from 0.248 mm at 9000 cycles to 0.990 and 1.915 mm at 10,000 and 11,000 cycles, respectively, and then increased markedly to 12.440 mm at 12,000 cycles, demonstrating the accelerated surface crack growth during the later fatigue stage.

#### 4.2.2. Evolution of Rayleigh-Wave Nonlinear Ultrasonic Response

Figure 9a presents the nonlinear parameter *β* measured by Rayleigh-wave tests as a function of fatigue cycles, and Figure 9b shows the normalized nonlinear parameter *β*′ obtained by normalizing *β* according to Equation (4). The Rayleigh-wave nonlinear parameter exhibits a distinct non-monotonic evolution that can be clearly divided into three characteristic stages.

During the 6000–8000 cycle interval (Stage I), the normalized nonlinear parameter increases continuously. Notably, no macroscopic crack is optically observable during this stage, yet the nonlinear parameter already exhibits a clear rising trend. This phenomenon indicates that nonlinear ultrasonic techniques can perceive microstructural changes within the material—such as dislocation multiplication, persistent slip band formation, and microvoid nucleation—before crack formation, even though these changes remain below the resolution of optical observation. The rise in the nonlinear parameter prior to macroscopic crack appearance endows it with unique advantages for early damage warning.

At 9000 cycles (Stage II), the normalized nonlinear parameter reaches its peak value, which coincides precisely with the first optical observation of a macroscopic crack (Figure 8c). This synchronism carries significant physical meaning: it indicates that the peak in the nonlinear parameter corresponds to the moment of crack initiation, when the crack faces undergo periodic opening and closure under ultrasonic excitation, generating strong contact nonlinearity and maximizing second-harmonic generation efficiency. According to the nonlinear spring model [27,36], the contact stiffness of the newly formed crack interface varies nonlinearly with the ultrasonic stress cycle, and its efficiency in harmonic generation is far higher than that of volume-distributed nonlinear sources such as dislocations, which is why the nonlinear parameter reaches its maximum at the moment of crack initiation.

During the 10,000–12,000 cycle interval (Stage III), the normalized nonlinear parameter decreases continuously from its peak. Although the crack continues to grow geometrically (Figure 8d), the nonlinear parameter decreases. This counterintuitive trend demonstrates that the nonlinear parameter is not a monotonic indicator of macroscopic crack length, but is governed by multiple factors including crack-interface contact conditions, crack opening displacement, and ultrasonic scattering attenuation. As the crack opening displacement increases, both the effective contact duration and the contact area between crack faces during ultrasonic excitation are reduced, weakening the contact-nonlinearity contribution to harmonic generation. Meanwhile, the increasing crack length makes the crack a more effective scatterer of ultrasonic waves, introducing additional attenuation to the propagating wave—particularly to the already weak second-harmonic component. The net result of these two effects is a continuous decrease in the nonlinear parameter.

#### 4.2.3. Mechanistic Interpretation of Rayleigh-Wave Nonlinear Response

Based on the experimental results presented above, this section provides a systematic mechanistic interpretation of the three-stage evolution of the Rayleigh-wave nonlinear parameter by combining the nonlinear spring model with previously reported fatigue-related nonlinear ultrasonic mechanisms.

Stage I (6000–8000 cycles): Nonlinearity enhancement dominated by distributed microdamage:

During the early stage of fatigue loading, although no optically detectable surface crack is observed, fatigue-induced microstructural evolution may already occur in the highly stressed region near the crack tip. Previous studies have demonstrated that cyclic deformation can induce the multiplication and rearrangement of dislocations and the formation of dislocation substructures, which can significantly enhance acoustic nonlinearity [44,45]. Cantrell and Yost showed that fatigue-induced dislocation dipole substructures can substantially increase the ultrasonic nonlinearity parameter [44]. More importantly, nonlinear ultrasonic measurements combined with TEM characterization of DA718 nickel-based superalloy revealed that the increase in acoustic nonlinearity was closely correlated with fatigue-induced banded dislocation structures and dislocation pile-ups [45].

The Rayleigh wave is confined to a near-surface layer of approximately one wavelength in depth (about 1.2 mm), and its propagation path directly interacts with the distributed microdamage in the near-surface region. These microdamage features constitute numerous microscopic nonlinear sources along the propagation path. Although the contribution of each individual source is weak, the continuous increase in their volumetric density leads to a progressively enhanced cumulative nonlinear effect, which is macroscopically manifested as a clear rising trend in the nonlinear parameter. Therefore, the nonlinear enhancement observed in Stage I is consistent with the progressive accumulation of fatigue-induced microstructural damage in the near-surface region.

Stage II (9000 cycles): Contact nonlinearity upon crack initiation:

As fatigue damage accumulates, the material gradually evolves from distributed microstructural damage toward microcrack initiation and subsequent crack growth. Previous nonlinear ultrasonic studies have shown that pronounced changes in acoustic nonlinearity can occur during this transition [46,47]. Kumar et al. demonstrated that nonlinear ultrasonic measurements can distinguish regimes associated with fatigue crack initiation, small-crack growth, and subsequent crack propagation [46], while Lyu et al. described the fatigue nonlinear response using a three-stage framework involving early dislocation evolution, microcrack initiation, and crack expansion [47].

The newly formed crack faces undergo periodic opening and closure (clapping) as well as tangential relative sliding (rubbing) under ultrasonic excitation, giving rise to a strongly nonlinear stress–displacement relationship. The Rayleigh-wave propagation path directly intersects the near-surface crack, rendering it highly sensitive to crack-interface contact nonlinearity. According to the nonlinear spring model, the contact stiffness of the crack interface varies nonlinearly with the ultrasonic stress cycle, and contributes to harmonic generation. The Rayleigh-wave nonlinear parameter reaches its maximum at approximately 9000 cycles, coinciding with the first optical observation of a surface crack. This correspondence is consistent with an increasing contribution of crack-related nonlinear interactions at this stage.

Stage III (9000–12,000 cycles): Competition between crack opening and scattering attenuation:

After crack initiation, the crack continues to propagate under further cyclic loading, and its opening state and interfacial contact conditions evolve continuously. Changes in crack opening can reduce the effective contact duration and contact area between crack faces during ultrasonic excitation, thereby weakening the crack-interface contribution to harmonic generation. Meanwhile, the increasing crack length makes the crack a more effective scatterer of ultrasonic waves, introducing additional attenuation to the propagating wave [48,49], particularly to the already weak second-harmonic component [50].

Similar non-monotonic nonlinear ultrasonic responses have been reported during fatigue crack evolution. Lyu et al. showed that the transmitted harmonic response decreases as fatigue damage evolves toward the crack-expansion stage [47], while recent nonlinear ultrasonic measurements on nickel-based superalloys have also reported a decrease in the nonlinear parameter during later stages of fatigue associated with microcrack propagation [8].

Therefore, the continuous decrease in the nonlinear parameter during Stage III may result from the combined evolution of crack-interface interaction and ultrasonic wave propagation as the crack grows.

In summary, the three-stage evolution of the Rayleigh-wave nonlinear parameter reflects a progressive transition in the dominant contributions to the nonlinear ultrasonic response, from volume-distributed microdamage in the early fatigue stage to increasingly important crack-interface nonlinear interactions during crack initiation and propagation, followed by the combined effects of evolving crack-interface conditions and ultrasonic scattering in the later stage. This mechanistic interpretation, supported by the nonlinear spring model and previous fatigue-related nonlinear ultrasonic studies, provides a physical basis for understanding the stage-dependent evolution of the nonlinear parameter during fatigue damage progression.

### 4.3. Comparison of Nonlinear Ultrasonic Responses Obtained Using Different Wave Modes

To assess the consistency of the observed nonlinear ultrasonic evolution and its dependence on measurement configuration, three distinct ultrasonic configurations were employed on the interrupted fatigue CT specimens: Rayleigh surface waves, normal-incidence longitudinal waves, and end-face-incidence longitudinal waves. These configurations differ in their propagation paths and wave–crack interaction geometries and therefore may provide complementary information on the evolution of fatigue damage and crack-related acoustic nonlinearity.

#### 4.3.1. Comparison of the Two Longitudinal-Wave Configurations

Figure 10a presents the normalized nonlinear parameters obtained from the normal-incidence and end-face-incidence longitudinal-wave configurations as a function of fatigue cycles. Both configurations exhibit a non-monotonic three-stage evolution—increase, peak, and decrease—but show different peak positions. The normal-incidence configuration reaches its maximum at 8000 cycles, whereas the end-face-incidence configuration peaks at 9000 cycles. Notably, the normal-incidence nonlinear parameter has already decreased by 9000 cycles, whereas the end-face-incidence configuration reaches its maximum at this fatigue state. This difference suggests that the two measurement configurations respond differently to the evolving fatigue-damaged and crack regions.

The difference in peak position may be interpreted in terms of the different wave–crack interaction geometries of the two configurations. In the end-face-incidence configuration (Figure 5b), the ultrasonic wave propagates along the specimen length direction, with its wave vector approximately normal to the crack plane. This propagation geometry allows the incident wave to interact more directly with the crack interface along its propagation path and may therefore enhance the contribution of crack-related nonlinear interactions to the measured response. The nonlinear parameter in this configuration reaches its maximum at 9000 cycles, when an optically detectable surface crack was first observed. The coincidence of these two observations suggests that the pronounced nonlinear response may be associated with changes in crack-interface conditions during the transition from the early fatigue-damage stage to the optically detectable crack-growth stage.

In contrast, in the normal-incidence configuration, the longitudinal wave propagates through the specimen thickness, approximately parallel to the crack plane (Figure 5a). Under this geometry, he interaction between the propagating wave and the crack interface differs from that in the end-face-incidence configuration. The measured nonlinear response may therefore contain different relative contributions from fatigue-induced microstructural changes, local stress and strain fields, and crack-related nonlinear interactions. During the early fatigue stage before an optically detectable surface crack is observed, fatigue-induced microstructural evolution and localized plastic deformation in the region near the notch root may contribute to the nonlinear response of the thickness-propagating longitudinal wave. These effects provide a plausible explanation for the earlier increase and peak observed in the normal-incidence configuration.

Specifically, during the 6000–8000 cycle interval, no optically detectable surface crack was observed, while the nonlinear parameter measured using the normal-incidence configuration increased progressively and reached its maximum at 8000 cycles. This increase may be associated with the accumulation of fatigue-induced microstructural changes and localized deformation in the region sampled by the ultrasonic wave. After 8000 cycles, the nonlinear parameter decreased despite continued fatigue loading and subsequent crack growth. As the crack develops, changes in the crack-interface condition, together with increasing wave scattering, diffraction, and transmission losses, may alter both the fundamental and second-harmonic components and consequently affect the measured nonlinear parameter. These combined effects provide a plausible explanation for the decrease observed after the peak. In comparison, the end-face-incidence configuration continued to exhibit an increasing nonlinear response up to 9000 cycles, which may be related to its different propagation direction and interaction with the evolving crack interface.

The different peak positions of the two longitudinal-wave configurations can therefore be interpreted as reflecting their different sensitivities to the evolving fatigue-damaged and crack regions. The earlier peak observed for the normal-incidence configuration may be associated with its response to fatigue-induced microstructural changes and localized deformation before an optically detectable surface crack is observed, whereas the later peak of the end-face-incidence configuration may reflect its stronger interaction with the evolving crack interface. At later fatigue stages, both configurations show decreasing nonlinear parameters and approach similar normalized levels. This convergence suggests that continued crack growth and the associated changes in crack-interface conditions and ultrasonic propagation progressively modify the nonlinear responses measured by both configurations.

#### 4.3.2. Comparison of Rayleigh Surface Waves and End-Face-Incidence Longitudinal Waves

Figure 10b compares the normalized nonlinear parameters obtained from Rayleigh surface-wave and end-face-incidence longitudinal-wave measurements. These two configurations are selected for comparison because both involve wave propagation geometries that allow interaction with the evolving crack region, although their propagation characteristics and sampling regions differ substantially. Rayleigh waves propagate along the specimen surface and are primarily sensitive to the near-surface region, whereas end-face-incidence longitudinal waves propagate along the specimen length direction and interact with the crack region along a different propagation path. These differences provide a useful basis for comparing their responses to fatigue-damage and crack evolution.

As shown in Figure 10b, both configurations exhibit a non-monotonic evolution characterized by an initial increase followed by a decrease, and their peak positions coincide at 9000 cycles. This common peak coincides with the fatigue state at which an optically detectable surface crack was first observed. The agreement between the two independently measured nonlinear responses suggests that the transition around 9000 cycles is associated with a pronounced change in the fatigue-damage and crack state. However, the coincidence of the nonlinear peaks with the first optical observation should not be interpreted as identifying the exact physical point of crack initiation, which may occur before the crack becomes optically detectable.

Nevertheless, notable differences exist in the detailed evolutionary features between the two configurations. The Rayleigh-wave nonlinear parameter exhibits a faster initial increase and consistently higher normalized values over the investigated fatigue-cycle range. Because Rayleigh-wave energy is concentrated near the specimen surface, its measured nonlinear response is expected to be more strongly influenced by fatigue-induced changes in the near-surface region containing the notch and developing surface crack. In contrast, the end-face-incidence longitudinal wave propagates through a larger material volume along the specimen length direction, so its measured response may contain contributions from both the evolving crack region and the surrounding material. These different propagation and sampling characteristics may contribute to the differences in the magnitudes and evolution rates of the normalized nonlinear parameters observed for the two configurations.

In the post-peak stage after 9000 cycles, both configurations exhibit continuous decreases in normalized nonlinear parameters, but the end-face-incidence configuration shows a more pronounced decline. As crack growth proceeds, changes in crack opening and interfacial contact conditions, together with the increasing influence of the growing crack on ultrasonic propagation and scattering, may modify the measured nonlinear response. Because the two configurations interact with the crack region through different propagation paths, the relative contributions of these effects may also differ. The Rayleigh-wave nonlinear parameter remains at a relatively higher normalized level during the later fatigue stages, suggesting that near-surface fatigue-damage and crack-related nonlinear interactions may continue to contribute to its measured response. The more pronounced decrease observed for the end-face-incidence configuration may similarly be associated with changes in crack-interface conditions and wave propagation along its measurement path. These observations indicate that the two configurations respond differently to the evolving crack state, although the individual contributions of crack-interface contact, scattering, and other propagation effects cannot be uniquely separated from the present measurements.

Based on the comprehensive comparison of the three measurement configurations (Figure 10a,b), several common and configuration-dependent features can be identified. First, all three configurations exhibit the same overall non-monotonic evolution—an increase followed by a peak and subsequent decrease—indicating that this trend is consistently observed across the different measurement configurations investigated in this study. Second, the peak position depends on the measurement configuration: the normal-incidence longitudinal-wave configuration peaks at 8000 cycles, whereas the Rayleigh-wave and end-face-incidence configurations both peak at 9000 cycles. These different peak positions are consistent with differences in propagation path and wave–damage/crack interaction geometry among the three configurations and suggest that each configuration may weight the evolving fatigue-damage and crack regions differently. Third, the normalized values of the Rayleigh-wave configuration are consistently higher than those of the end-face-incidence configuration throughout the investigated fatigue-cycle range. This difference may be related to the near-surface propagation characteristics of Rayleigh waves and their interaction with the fatigue-damaged and crack regions near the specimen surface; however, differences in normalized magnitude alone should not be interpreted as a direct quantitative measure of relative sensitivity between the two configurations. Fourth, the different rates of decline among the configurations during the later fatigue stages suggest that the measured nonlinear response is influenced by the combined effects of evolving crack-interface conditions and ultrasonic propagation characteristics as the crack grows.

These results also highlight the potential value of combining multiple ultrasonic measurement configurations for fatigue damage characterization. Because each configuration follows a different propagation path and interacts differently with the fatigue damaged and crack regions, measurements based on different wave modes can provide complementary information on the evolution of fatigue damage and crack propagation. In the present experiments, the earlier peak of the normal-incidence longitudinal-wave response and the later coincident peaks of the Rayleigh-wave and end-face-incidence responses demonstrate that the three configurations do not respond identically to the same fatigue state. Therefore, comparison among multiple configurations may assist in distinguishing different stages of fatigue damage evolution.

### 4.4. Comparison of Nonlinear and Linear Ultrasonic Responses

Conventional linear ultrasonic techniques assess material integrity primarily through changes in wave velocity, attenuation, reflection, scattering, and transmission caused by material degradation or defects. These methods are effective for detecting macroscopic defects with significant acoustic impedance mismatch, whereas their sensitivity to early-stage fatigue damage is generally more limited. This section compares the evolution of nonlinear ultrasonic parameters with that of linear ultrasonic parameters, including longitudinal-wave velocity and attenuation coefficient, during fatigue damage accumulation and crack propagation.

Figure 11a presents the evolution of longitudinal-wave velocity as a function of fatigue cycles. Throughout the entire fatigue process, the longitudinal-wave velocity exhibits only small fluctuations around approximately 5840 m/s, without a clear monotonic or stage-dependent trend. These results suggest that the fatigue-induced changes in the effective elastic properties are insufficient to produce a pronounced variation in the measured longitudinal-wave velocity. Therefore, under the present experimental conditions, longitudinal-wave velocity exhibits relatively low sensitivity to the evolution of fatigue damage.

Figure 11b shows the evolution of the ultrasonic attenuation coefficient with fatigue cycles. From 6000 to 10,000 cycles, the attenuation coefficient fluctuates without a clear monotonic trend. From 11,000 cycles onward, a slight increasing tendency is observed. This change occurs during the later stage of crack propagation and may be associated with the increasing influence of the growing crack on ultrasonic scattering and transmission. Nevertheless, the overall variation in attenuation remains relatively small compared with the pronounced evolution of the nonlinear ultrasonic parameter.

Overall, the linear ultrasonic parameters (velocity and attenuation coefficient) do not exhibit any characteristic changes during the early and intermediate stages of fatigue (6000–9000 cycles), and the attenuation coefficient only shows a weak response in the later stage of fatigue after substantial crack propagation. This indicates that, under the present fatigue loading conditions and crack propagation scale, the changes in effective elastic and wave-propagation properties of the material are relatively limited, and linear ultrasonic parameters exhibit low sensitivity to early-stage fatigue damage and the transition toward crack development.

In contrast, the nonlinear ultrasonic parameter exhibits a pronounced non-monotonic three-stage evolution: it begins to rise continuously during 6000–8000 cycles, reaches its peak at 9000 cycles, and then gradually decreases. The linear ultrasonic parameters, however, exhibit only relatively small variations over the same fatigue stages, with their fluctuation amplitudes far smaller than the variation range of the nonlinear parameter. This comparison indicates that, under the present experimental conditions, nonlinear ultrasonic characterization is more sensitive to fatigue damage evolution than the investigated conventional linear ultrasonic parameters.

The fundamental difference in sensitivity between linear and nonlinear ultrasonic methods originates from their distinct physical mechanisms of response to material damage. Linear ultrasonic parameters primarily reflect changes in macroscopic physical properties such as elastic modulus and density, as well as changes in ultrasonic propagation caused by defects such as cracks and voids. Only when microstructural changes induced by fatigue damage become sufficiently significant to cause measurable alterations in the effective elastic and wave-propagation properties do linear ultrasonic parameters exhibit appreciable variation. The experimental results of this study indicate that, at the early and intermediate stages of fatigue, such changes are insufficient to produce pronounced variations in the measured linear ultrasonic parameters; the attenuation coefficient only exhibits a weak response after substantial crack propagation.

In contrast, nonlinear ultrasonic parameters are highly sensitive to crack-interface contact conditions and local nonlinear interactions associated with fatigue-induced microstructural changes. Ultrasonic nonlinear effects originate from the nonlinear interaction between the ultrasonic wave and the internal microstructure of the material. In the early stage of fatigue damage, fatigue-induced evolution of dislocation structures and other microstructural damage can enhance acoustic nonlinearity even when the corresponding changes in macroscopic elastic properties remain limited. As fatigue damage evolves toward crack initiation and propagation, crack-related nonlinear interactions may become increasingly important. The nonlinear spring model indicates that periodic opening and closing of interacting crack faces under ultrasonic excitation can produce strong contact nonlinearity and contribute significantly to harmonic generation. In the present study, the nonlinear parameter reaches its maximum at approximately 9000 cycles, around the first optical observation of a surface crack, which is consistent with an increasing contribution of crack-related nonlinear interactions at this stage. In the later stage of crack propagation, despite continued crack growth, the evolving crack opening state and interfacial contact conditions may modify the crack-related contribution to harmonic generation, while the increasing influence of the growing crack on ultrasonic propagation and scattering may further affect the measured nonlinear response. The nonlinear ultrasonic parameter reflects the cumulative effect of wave interaction with various nonlinear sources along the propagation path, which explains its greater sensitivity to fatigue-induced microstructural and crack-related changes compared with the investigated linear ultrasonic parameters.

The above comparison results carry important engineering implications. For fatigue state assessment of in-service structural materials, relying solely on linear ultrasonic parameters may be insufficient for effectively identifying early-stage fatigue damage and comprehensively monitoring crack evolution. Nonlinear ultrasonic techniques, owing to their sensitivity to microstructural changes and crack-interface conditions, can provide complementary information for early fatigue-damage detection and crack-evolution monitoring. Of particular significance is the fact that the nonlinear parameter exhibits a measurable rising trend before the first optical observation of a surface crack (6000–8000 cycles), which demonstrates its potential advantages in early fatigue-damage assessment and structural health monitoring. In practical engineering applications, nonlinear ultrasonic testing may therefore serve as an effective complement to conventional linear ultrasonic testing, providing additional information for characterizing the fatigue damage process from early microstructural changes to subsequent crack propagation.

## 5. Conclusions

This study systematically investigated the evolution of nonlinear ultrasonic responses during fatigue damage accumulation and crack propagation in GH4169 nickel-based superalloy, combining controlled-crack experiments on laser-fabricated specimens with interrupted fatigue tests on compact-tension (CT) specimens. Three distinct ultrasonic wave modes—Rayleigh surface waves, normal-incidence longitudinal waves, and end-face-incidence longitudinal waves—were employed to provide complementary characterization, and the results were compared with conventional linear ultrasonic parameters. The following conclusions can be drawn:

(1) The laser-fabricated controlled-crack experiments showed a crack geometry dependence consistent with that predicted by the nonlinear spring model. The normalized nonlinear parameter *β*′ exhibits excellent linear correlations with *a*^2^ and 1/*b*^2^, in strong agreement with the theoretical prediction of *β* ∝ *a*^2^/*b*^2^. The results support the predicted relationship between crack geometry and nonlinear ultrasonic response, although the laser-fabricated cracks do not fully reproduce the dynamic contact behavior of actual fatigue cracks.

(2) The Rayleigh-wave nonlinear parameter exhibits a distinct non-monotonic three-stage evolution during fatigue crack propagation: a continuous increase during 6000–8000 cycles, a peak at 9000 cycles when an optically detectable surface crack was first observed, and a subsequent decrease during 10,000–12,000 cycles. The increase before the first optical detection of a surface crack indicates the sensitivity of the nonlinear parameter to early fatigue damage, while the post-peak decrease despite continued crack growth reveals that the nonlinear response is not a simple monotonic indicator of crack length but is also sensitive to the evolving fatigue-damage and crack state.

(3) All three ultrasonic configurations exhibit similar overall non-monotonic evolution trends, although their peak positions differ. The normal-incidence longitudinal-wave configuration peaks at 8000 cycles, whereas the Rayleigh-wave and end-face-incidence configurations peak at 9000 cycles. These differences may be associated with their different propagation paths and interactions with the evolving damaged and cracked regions. In contrast, the linear ultrasonic parameters show relatively limited changes during the early fatigue stages, indicating the complementary sensitivity of nonlinear ultrasonic measurements to early fatigue damage under the present experimental conditions.

(4) The three-stage nonlinear evolution can be interpreted as the combined result of fatigue-induced microstructural evolution, crack-related nonlinear interactions, and changes in ultrasonic propagation and scattering during crack growth. These mechanisms provide a plausible physical interpretation of the experimental observations rather than uniquely identified contributions. This framework provides a theoretical basis for understanding the physical significance of the nonlinear parameter in fatigue assessment and offers guidance for developing multi-mode ultrasonic condition monitoring strategies for critical aerospace components.

## Figures and Tables

**Figure 1:**
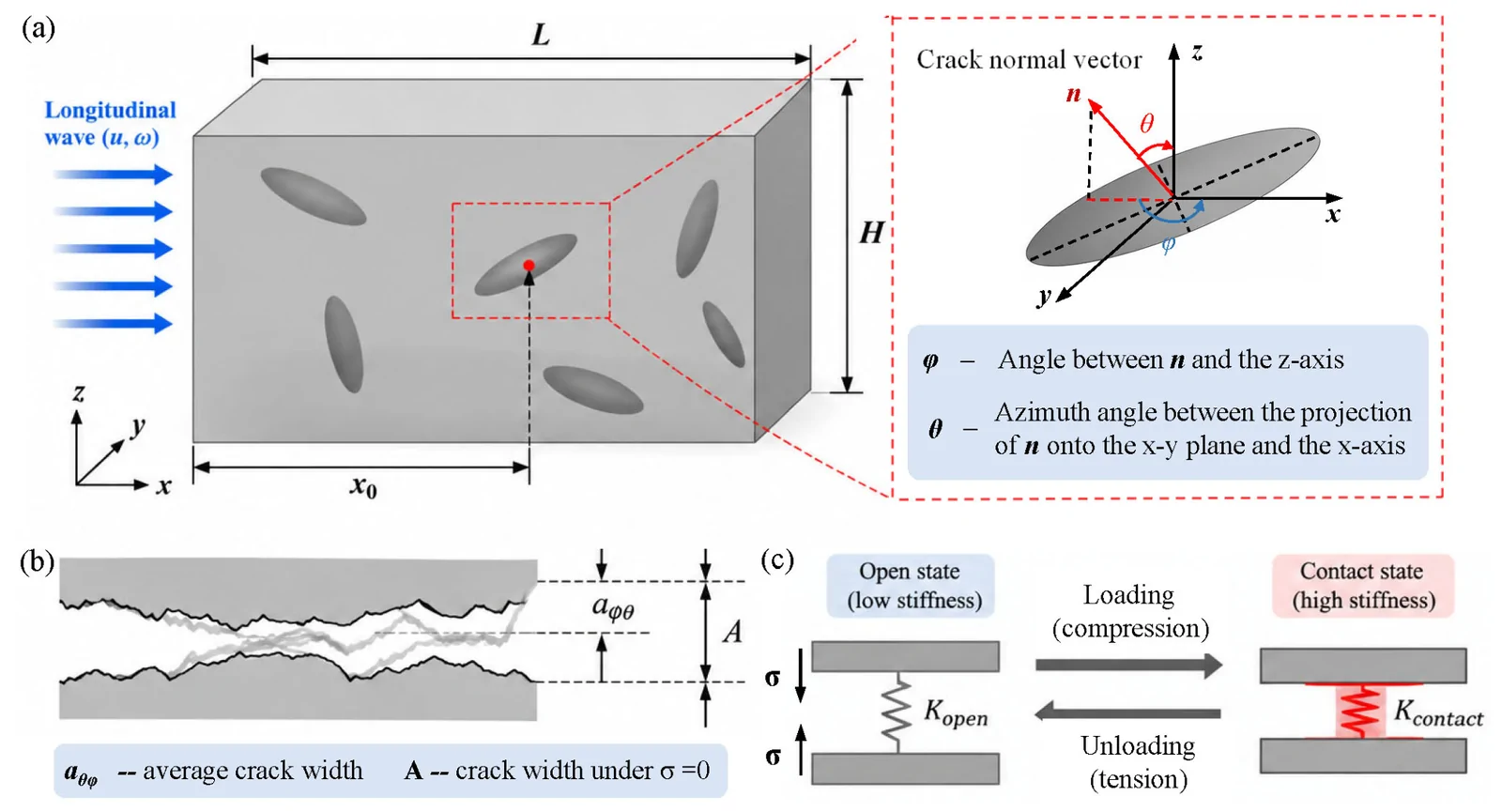
Schematic illustration of the microcrack nonlinear spring model: (**a**) Location and orientation of a planar microcrack in a metal plate under longitudinal-wave excitation; (**b**) rough crack interface and crack opening under applied stress; (**c**) Nonlinear spring representation of the crack opening–contact behavior during loading and unloading.

**Figure 2:**
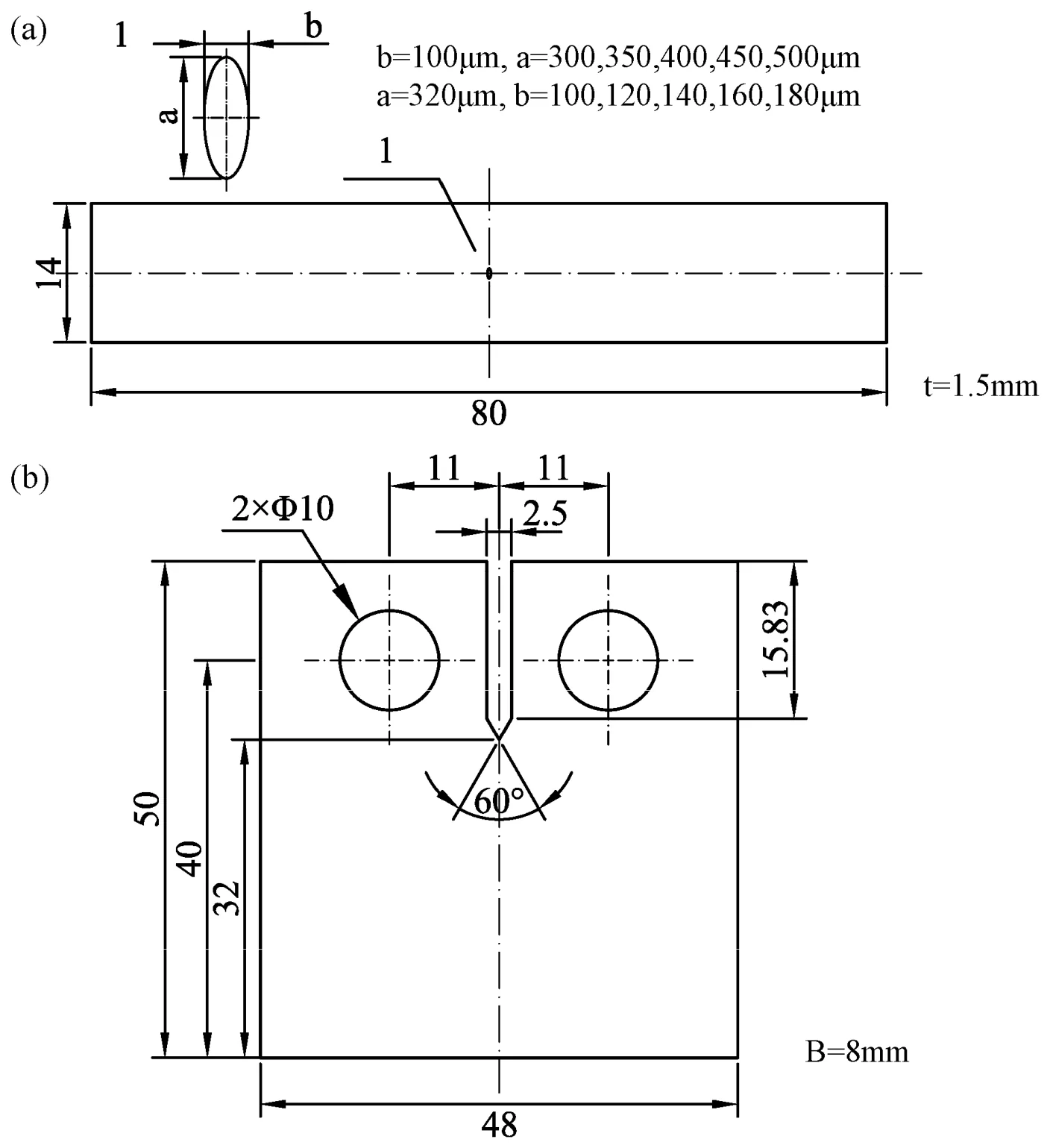
Geometric dimensions of Specimens (units: mm): (**a**) Laser-fabricated crack specimens; (**b**) CT specimens.

**Figure 3:**
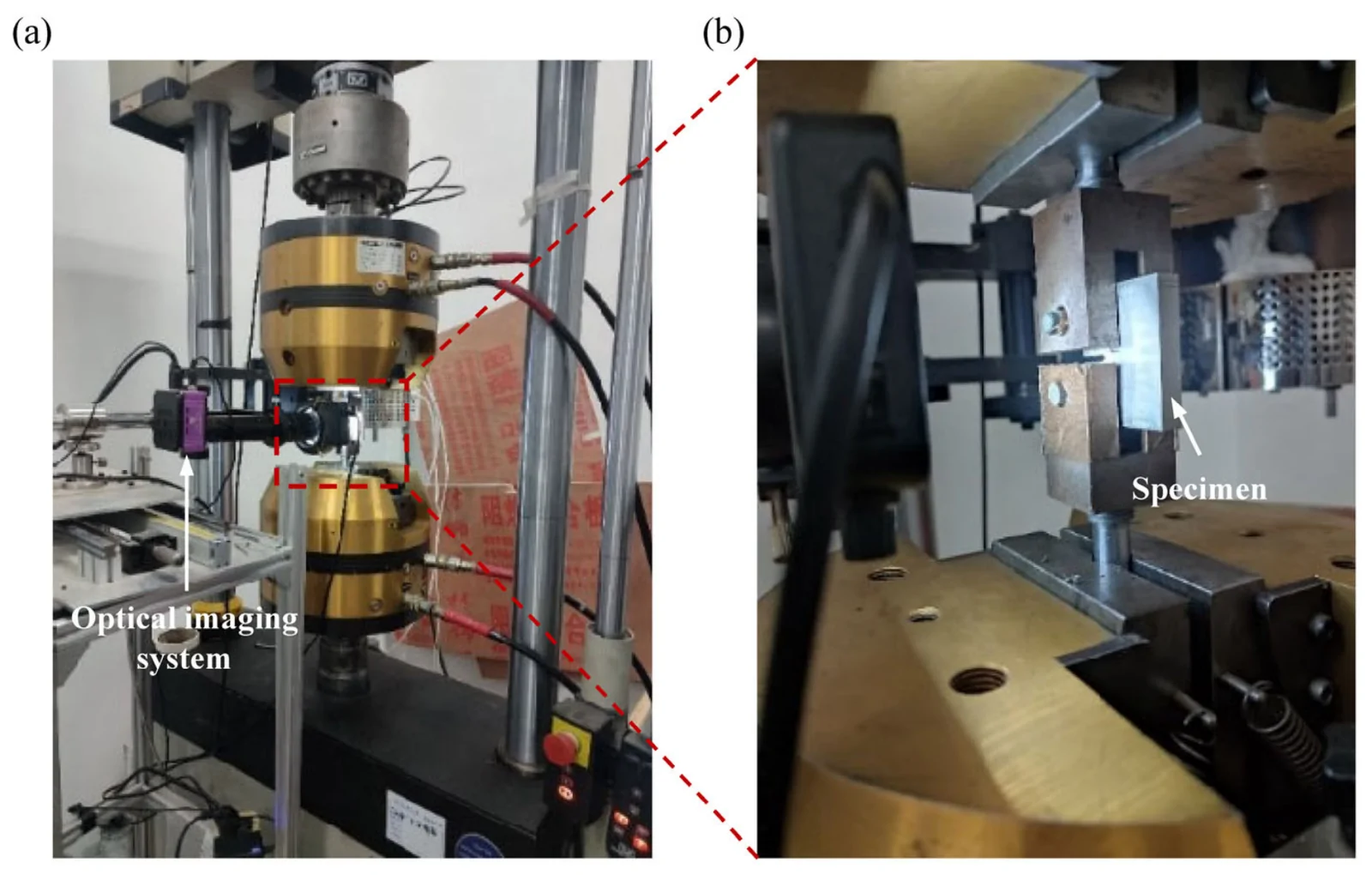
Fatigue test system: (**a**) Electro-hydraulic servo fatigue testing machine; (**b**) Partial magnification of specimen clamping region.

**Figure 4:**
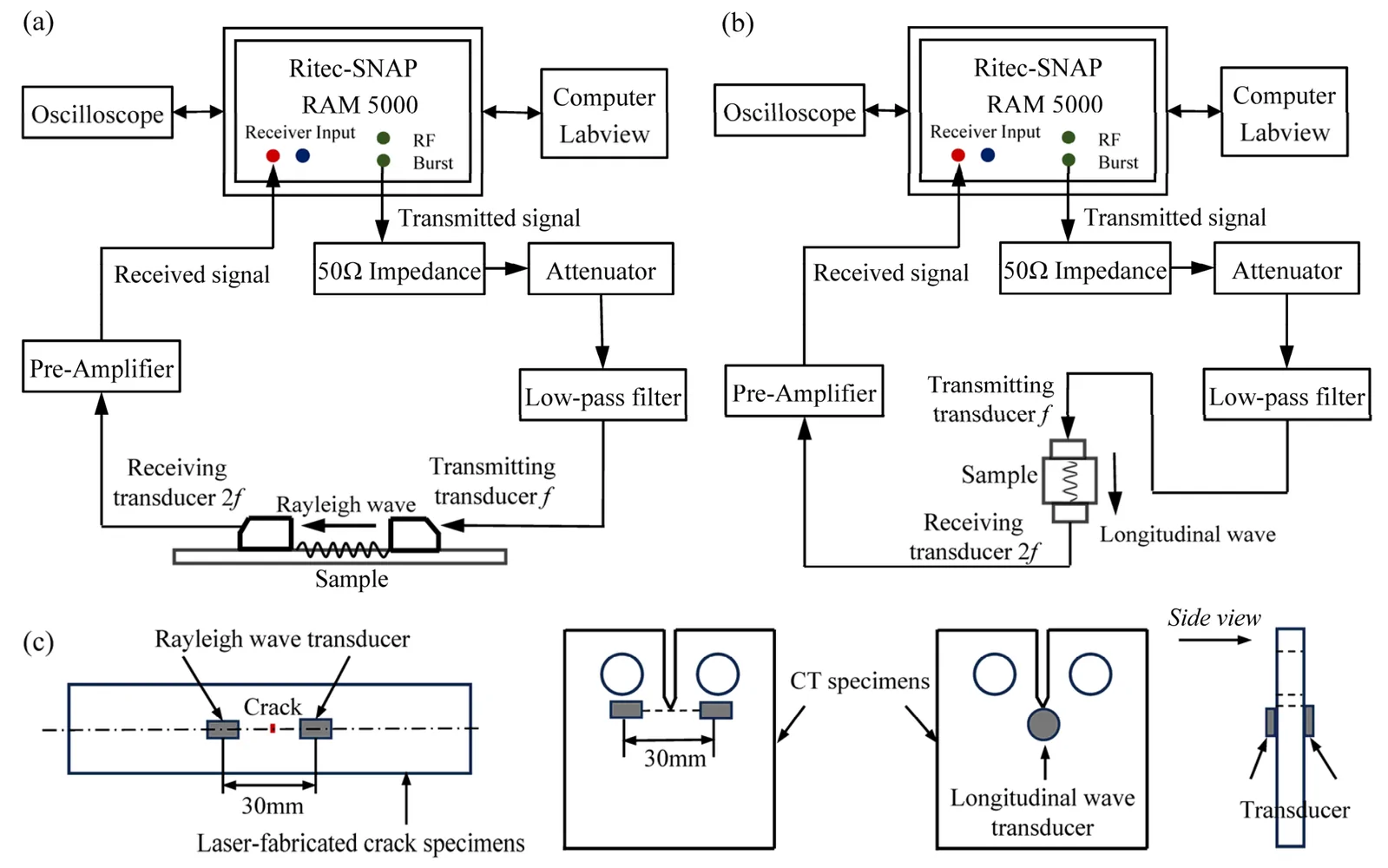
Non-linear ultrasonic measurement system: (**a**) Non-linear Rayleigh wave testing system; (**b**) Non-linear longitudinal wave testing system; (**c**) Schematic diagram of nonlinear ultrasonic detection position.

**Figure 5:**
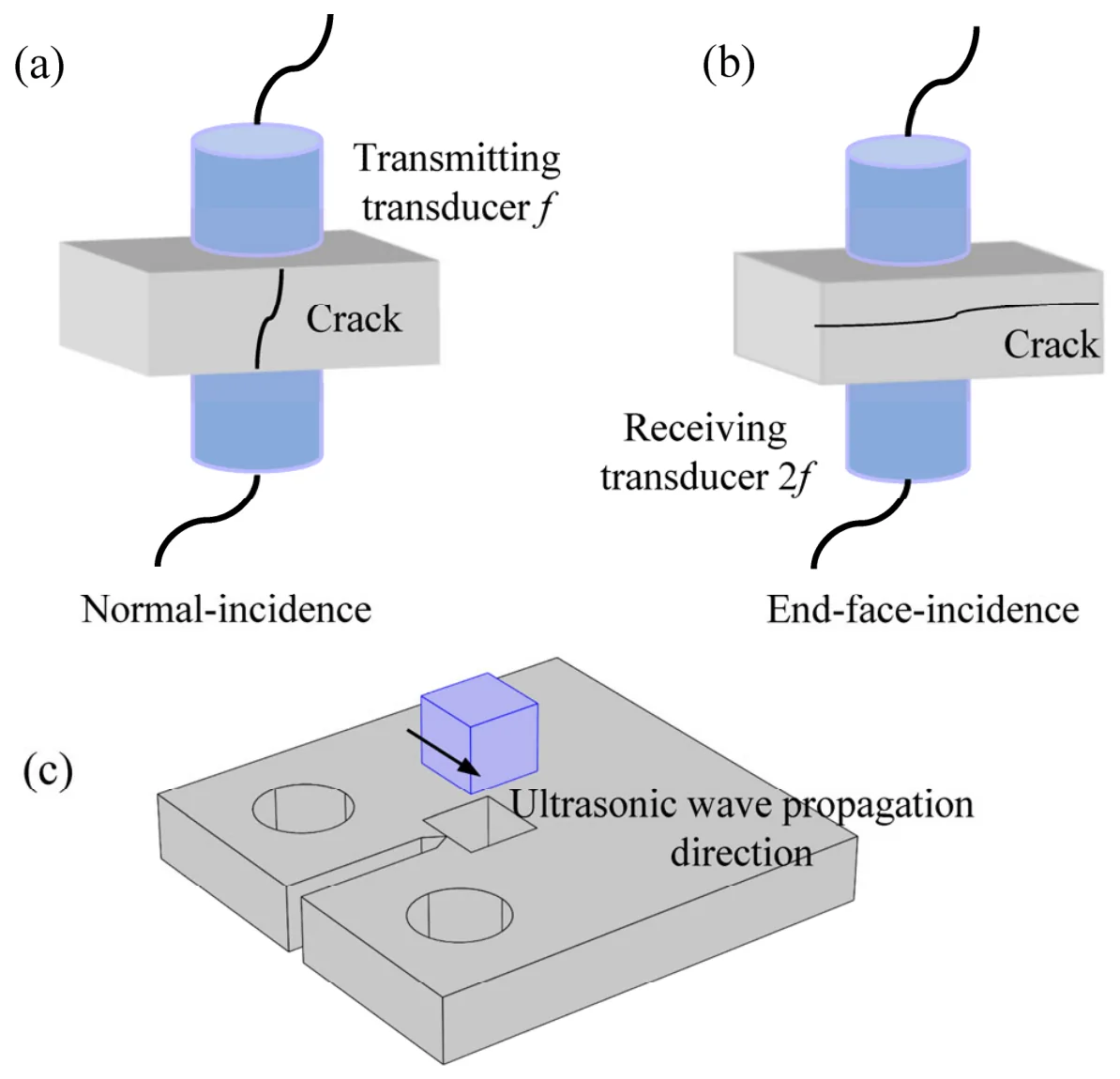
Schematic diagrams and specimens for end-face-incidence and normal-incidence measurements: (**a**) Schematic of the normal-incidence configuration; (**b**) Schematic of the end-face-incidence configuration; (**c**) Schematic of the end-face-incidence specimen together with the wave propagation direction.

**Figure 6:**
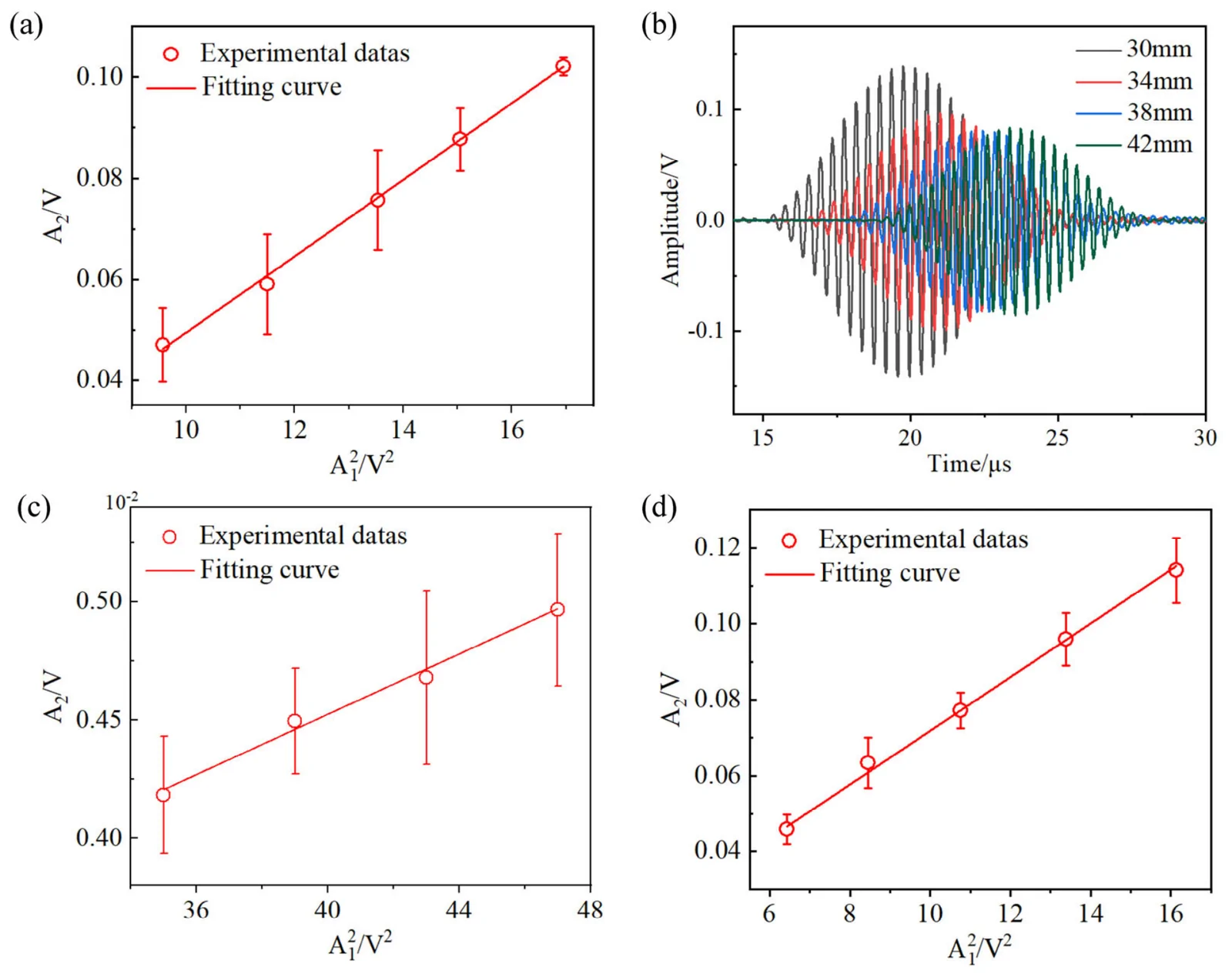
Calibration of nonlinear measurement system: (**a**) Voltage calibration of Rayleigh-wave detection system; (**b**) Time-domain signal for Rayleigh-wave system distance calibration; (**c**) Distance calibration of Rayleigh-wave system; (**d**) Voltage calibration of longitudinal-wave system. Data points represent the mean of five repeated measurements, and the error bars indicate ±1 standard deviation (SD).

**Figure 7:**
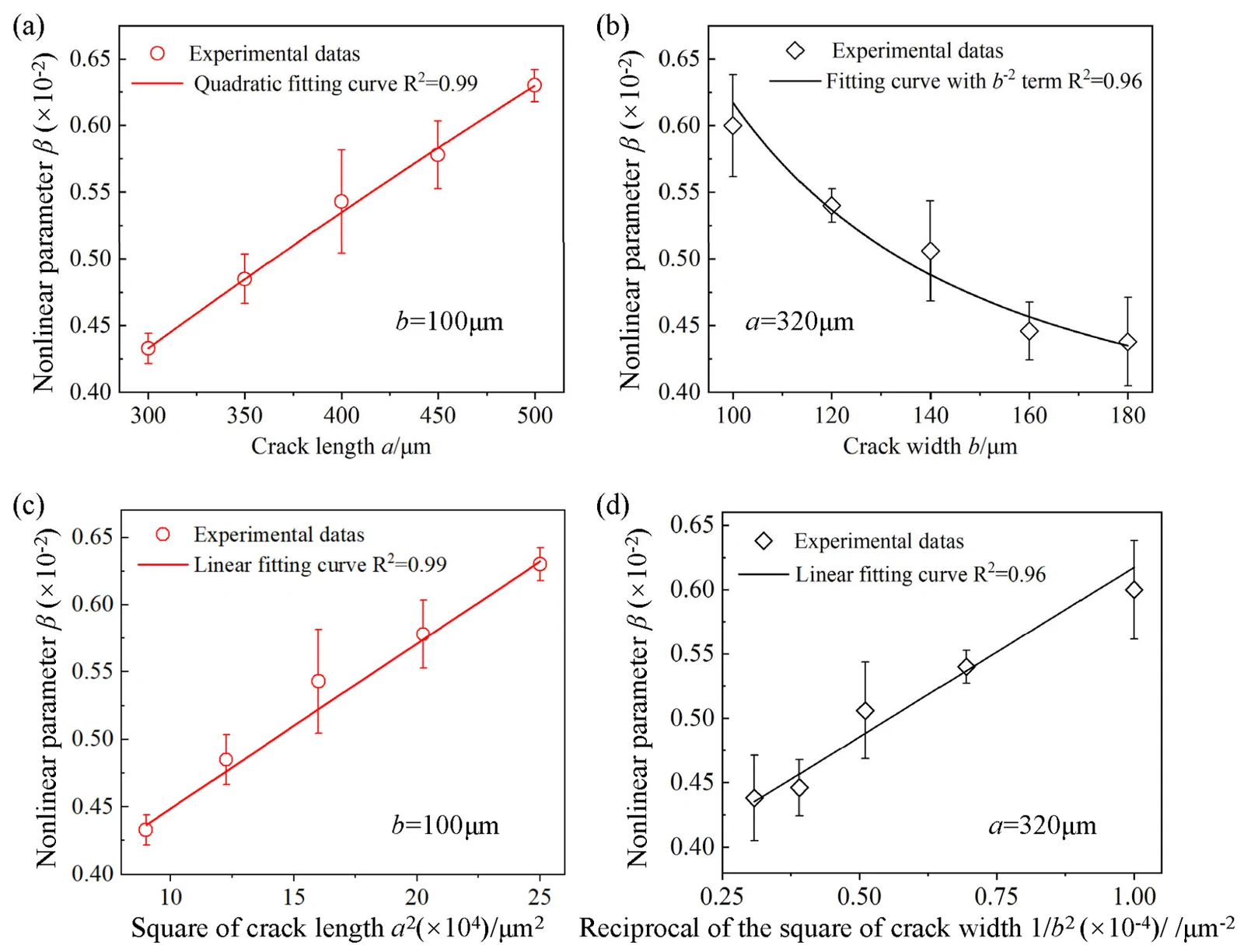
Relationship between nonlinear parameter and crack geometric dimensions: (**a**) Ultrasonic nonlinear parameter versus crack length; (**b**) Ultrasonic nonlinear parameter versus the reciprocal of the square of crack width; (**c**) Ultrasonic nonlinear parameter versus the square of crack length; (**d**) Ultrasonic nonlinear parameter versus the reciprocal of the square of crack width.

**Figure 8:**
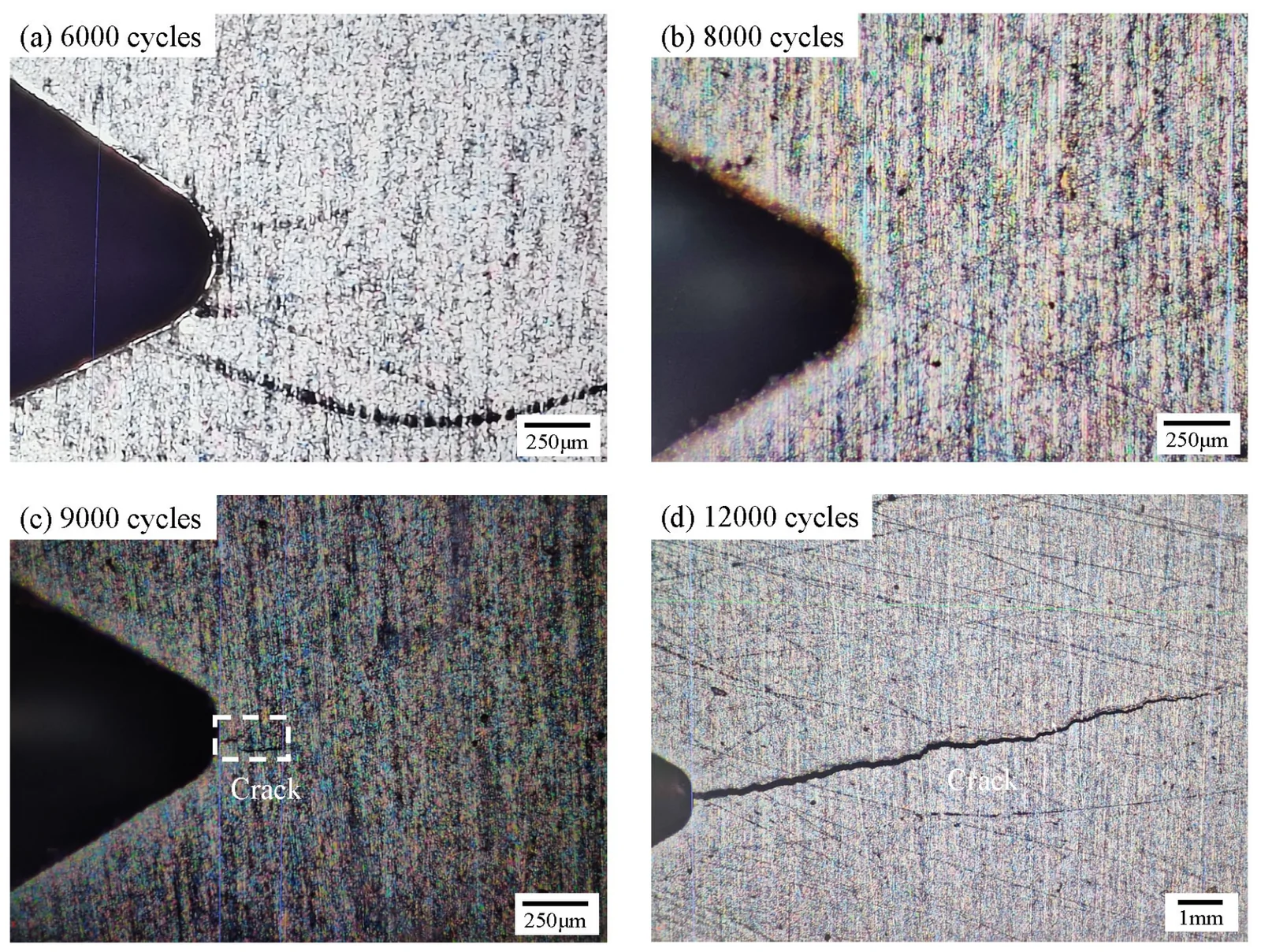
Optical micrographs showing the evolution of fatigue-crack morphology in GH4169 CT specimens at different fatigue-cycle numbers: (**a**) 6000 cycles; (**b**) 8000 cycles; (**c**) 9000 cycles; (**d**) 12,000 cycles.

**Figure 9:**
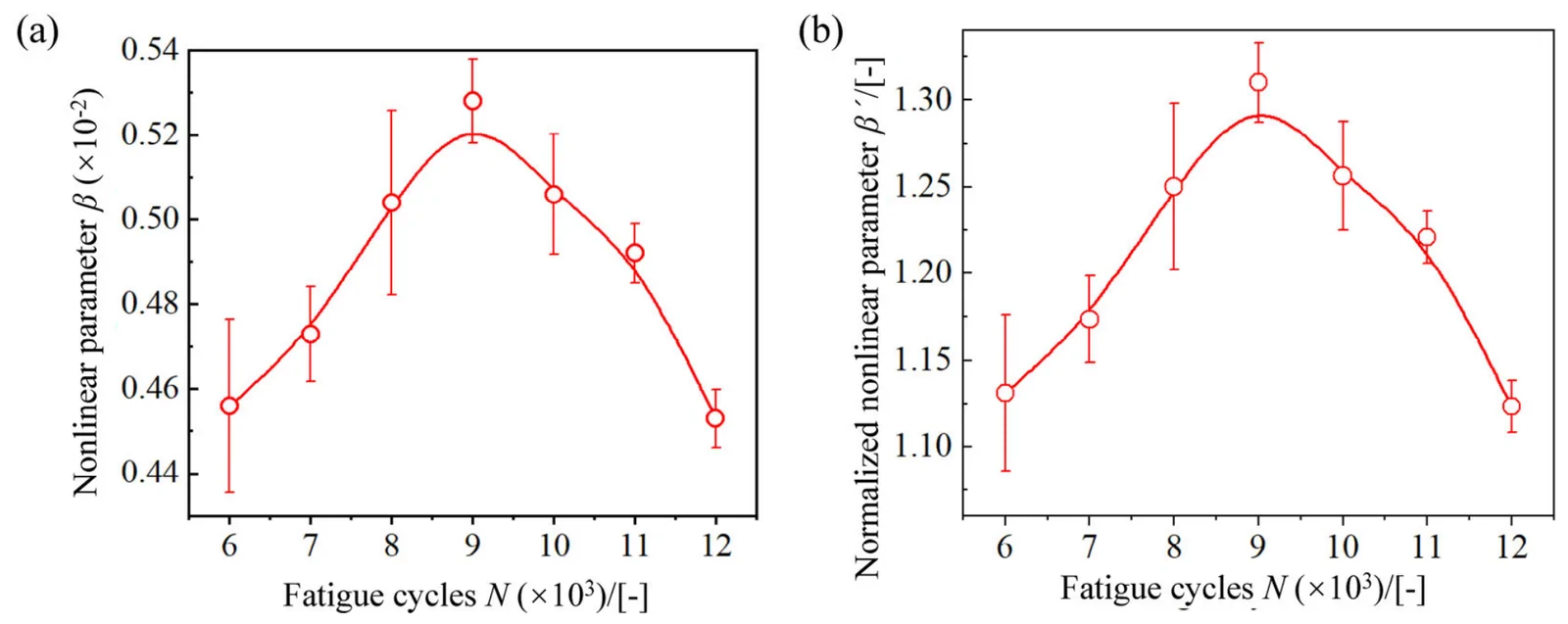
Evolution of Rayleigh-wave ultrasonic nonlinear parameter during fatigue: (**a**) Rayleigh-wave ultrasonic nonlinear parameter vs. fatigue cycles; (**b**) Normalized Rayleigh-wave ultrasonic nonlinear parameter vs. fatigue cycles.

**Figure 10:**
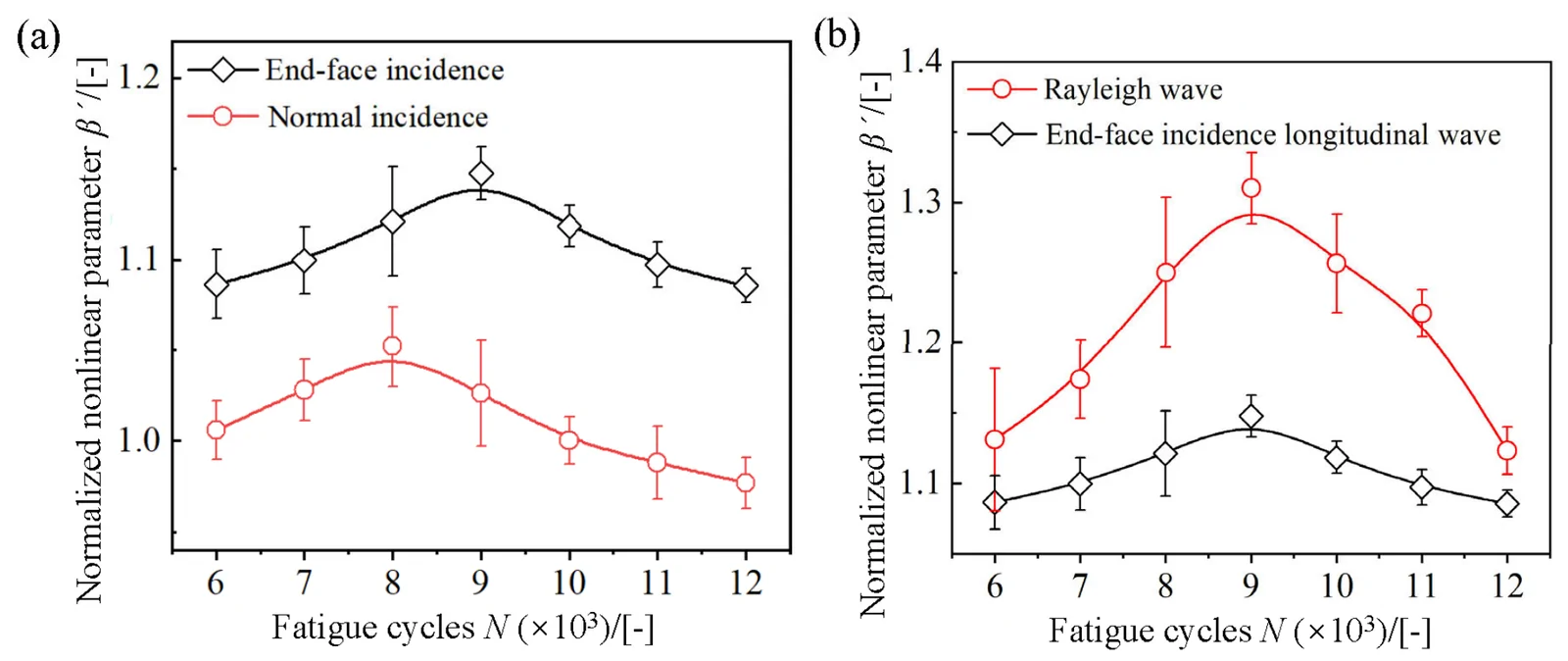
Comparison of normalized ultrasonic nonlinear parameters measured by different wave modes: (**a**) Comparison of normalized ultrasonic nonlinear parameters for normal-incidence and end-face-incidence longitudinal-wave configurations; (**b**) Comparison of normalized ultrasonic nonlinear parameters between Rayleigh-wave and end-face-incidence longitudinal-wave.

**Figure 11:**
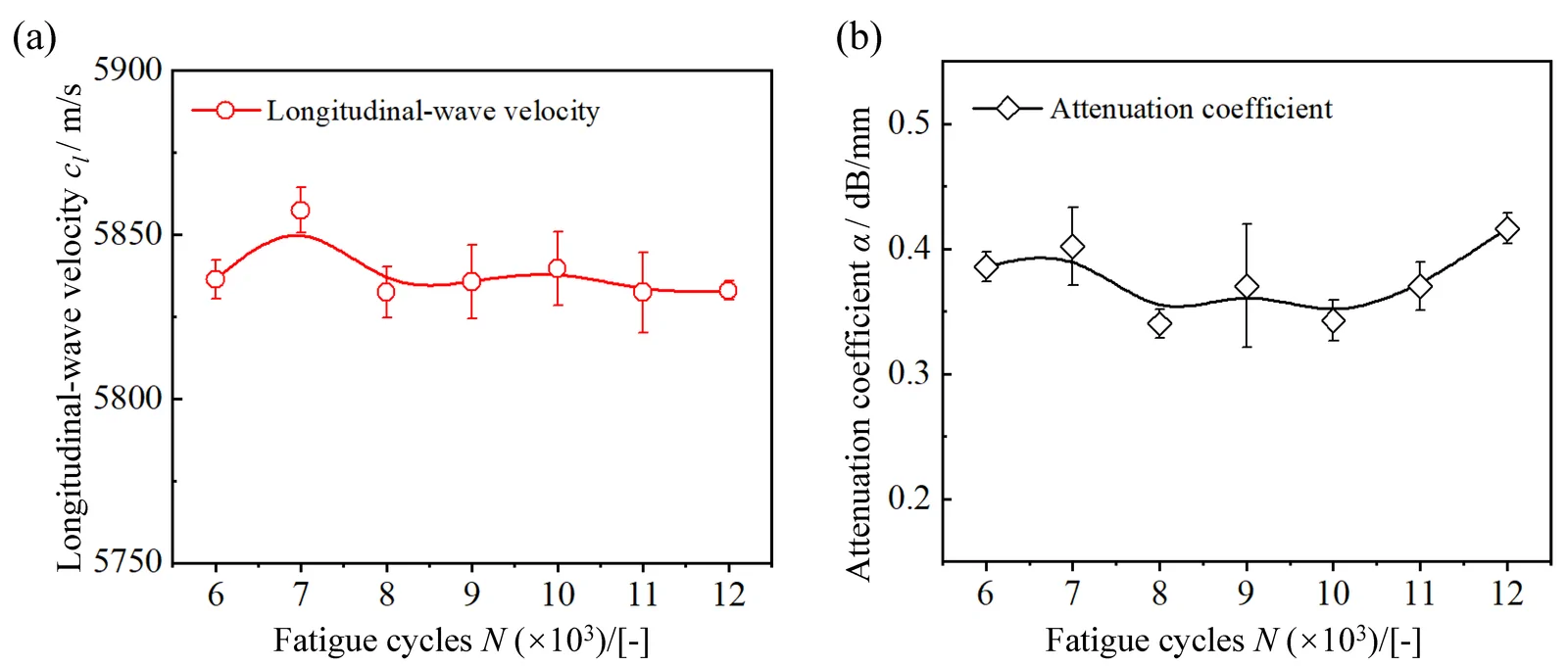
Evolution of linear ultrasonic parameters with fatigue cycles: (**a**) Longitudinal-wave velocity; (**b**) Ultrasonic attenuation coefficient.

**Table 1:** Nominal chemical composition of GH4169 superalloy (in wt.%).

Element	Ni	Cr	Nb	Mo	Ti	Si	Mn	Al	C	Fe
Content	53.0	19.6	6.0	3.0	0.95	0.35	0.35	0.25	0.08	Bal.

**Table 2:** Main mechanical-property parameters of GH4169.

Property	E/GPa	ν	G/GPa	σ_s_/MPa	σ_b_/MPa
Value	204	0.3	79	1080	1340

**Table 3:** Geometrical parameters of the laser-fabricated cracks.

Experimental Series	Fixed Parameter	Variable Parameter	Crack Dimensions/μm
Crack-length effect	*b* = 100 μm	Crack length *a*	300, 350, 400, 450, 500
Crack-width effect	*a* = 320 μm	Crack width *b*	100, 120, 140, 160, 180

**Table 4:** Optically measured surface crack extension at different fatigue-cycle conditions.

Fatigue Cycles, *N*	9000	10,000	11,000	12,000
Crack extension, ∆*a* (mm)	0.248	0.990	1.915	12.440

## Data Availability

The original contributions presented in this study are included in the article. Further inquiries can be directed to the corresponding author.
